# Supplementary material for: Dynamic and transformable Cu12 cluster-based C-H···π-stacked porous supramolecular frameworks
Source: Nat Commun. 2023 Oct 12;14:6413. doi: 10.1038/s41467-023-42201-w (PMC10570389; doi:10.1038/s41467-023-42201-w)
Supplement: Supplementary file 1 — Supplementary Information [file 41467_2023_42201_MOESM1_ESM.pdf]

Supplementary Information

Dynamic and transformable Cu_{12} cluster-based C-H···π-stacked porous supramolecular frameworks

Chengkai Zhang,[1] Zhi Wang,[1] Wei-Dan Si,[1] Hongxu Chu,[1] Lan Zhou,[1] Tong Li,[1] Xian-Qiang Huang,[2] Zhi-Yong Gao,[3] Mohammad Azam,[4] Chen-Ho Tung,[1] Ping Cui,[1] and Di Sun[1,*]

[1]School of Chemistry and Chemical Engineering, State Key Laboratory of Crystal Materials, Shandong University, Ji'nan 250100, People's Republic of China.

[2]Shandong Provincial Key Laboratory of Chemical Energy Storage and Novel Cell Technology, and School of Chemistry and Chemical Engineering, Liaocheng University, Liaocheng 252000, People's Republic of China.

[3]School of Chemistry and Chemical Engineering, Henan Normal University, Xinxiang, 453007, People's Republic of China.

[4]Department of Chemistry, College of Science, King Saud University, P. O. Box 2455, Riyadh 11451, Saudi Arabia.

[*]To whom correspondence should be addressed.

E-mail: dsun@sdu.edu.cn.

Section 1. Supplementary Methods

I. Materials and reagents

All chemicals and solvents used in the syntheses were of analytical grade and used without further purification.

II. Infrared spectroscopy

Infrared spectra were recorded on a PerkinElmer Spectrum Two in the frequency range of 4000-500 cm^{-1}.

III. Powder X-ray diffraction

Powder X-ray diffraction (PXRD) analyses were carried out on a microcrystalline powder using a Rigaku Oxford Diffraction XtaLAB Synergy-S diffractometer using Cu radiation (= 1.54184 Å). The PXRD patterns were processed with the CrysAlis^{Pro} software suite using the Powder function.

IV. UV-Vis absorption spectroscopy

The diffuse-reflectance spectra were performed on UV-Vis spectrophotometer (Evolution 220, ISA-220 accessory, Thermo Scientific) using a built-in 10 mm silicon photodiode with a 60 mm Spectralon sphere.

V. Scanning electron microscope and elemental mapping images

Morphology of the samples and elemental composition analyses were measured using an SU-8010 field emission scanning electron microscope (FESEM; Hitachi Ltd., Tokyo, Japan) equipped with an Oxford-Horiba Inca XMax50 energy dispersive X-ray spectroscopy (EDS) attachment (Oxford Instruments Analytical, High Wycombe, England).

VI. Gas adsorption measurements.

The adsorption isotherms of N_2, CO_2, CH_4, C_2H_4 and C_2H_6 were measured on Micromeritics (ASAP 2460) apparatus. Prior to gas uptake measurements, as-synthesized samples were solvent-exchanged over 24 h. And then the solvent was removed and the sample was dried under high vacuum at room temperature for 12 h.

VII. Thermogravimetic analysis (TGA)

Thermogravimetic analysis (TGA) was done in a TA SDT Q600 thermal analyzer at a

heating rate of 20°C min^{-1} under N_2 atmosphere (200 mL·min^{-1}) from 20 to 800°C.

VIII. X-ray Crystallography

Single crystals of **Cu12a**, **Cu12a-π**, **Cu12a-readsorption**, **Cu12b** and **Cu12b-NACs** with appropriate dimensions were chosen under an optical microscope and quickly coated with high vacuum grease (Dow Corning Corporation) to prevent decomposition. Crystals were mounted on CryoLoop™ loop and the cell parameters and intensity data were recorded on a Rigaku Oxford Diffraction XtaLAB Synergy diffractometer equipped with a HyPix-6000HE Hybrid Photon Counting (HPC) detector operating in shutterless mode and an Oxford Cryosystems Cryostream 800 Plus using Cu Kα (λ = 1.54184 Å) for **Cu12a**, **Cu12a-π**, **Cu12a-readsorption**, **Cu12b** and **Cu12b-NACs** from PhotonJet micro-focus X-ray Source at 100/173 K. Data were processed using the *CrystAlis*^{Pro} software suite.[1] These structures were solved using the charge-flipping algorithm, as implemented in the program *SUPERFLIP*[2] and refined by full-matrix least-squares techniques against F_o^2 using the SHELXL program[3] through the OLEX2 interface.[4] Hydrogen atoms at carbon were placed in calculated positions and refined isotropically by using a riding model. Appropriate restraints or constraints were applied to the geometry and the atomic displacement parameters of the atoms in the cluster. All structures were examined using the Addsym subroutine of PLATON[5] to ensure that no additional symmetry could be applied to the models. Pertinent crystallographic data collection and refinement parameters are collated in Table S1. Selected bond lengths and angles are collated in Table S2.

IX. Computational Studies

DFT calculation details: DFT and single point energy calculations were performed with the Gaussian 16 suite of programs.[6] For the optimizations of the cationic clusters **Cu12a** and **Cu12b** the gradient-corrected PBE1PBE exchange correlation functional, based on the generalized gradient approximation (GGA) was utilized; LanL2DZ (Los Alamos effective core potential double) basis set was employed for Cu atoms, and 6-31G* basis set was used for C, H, O, S and N atoms. The PBE1PBE functional is expected to be more suitable for the transition metal systems. The LANL2DZ basis set

containing relativistic effects has been shown to predict accurately the structure of Cu nanoclusters. Spin-restricted calculations were used for geometry optimization. Harmonic frequencies were then calculated to characterize the stationary points as equilibrium structures with all real frequencies, and to evaluate zero-point energy (ZPE) corrections. The TD-DFT calculations were performed to get the most probable transitions and the orbitals corresponding to the main peaks in the calculated electronic spectrum. A total of 300 singlet states were chosen in the calculations. The root is set as 1 in the TD-DFT calculations. Data for orbital composition analysis with Mulliken partition are from Gaussian 16 calculations and further processed with Multiwfn software.[7] The most probable transitions were determined based on the oscillator strength values and weights. The optical absorption spectra were convoluted with a Gaussian line shape with a half-width at half-height of 0.20 eV.

Energies calculation details: The energy calculations were carried out with the Gaussian 16 programs. The transformation model was established as follow: Cu12a + 2(PTC4A)→Cu12b + 2(TC4A). To minimize the calculation error, we combine the cluster and introduced ligand into one computational input file (i.e., [Cu12a + 2(PTC4A)].gjf or [Cu12b + 2(TC4A)].gjf) to calculate the electronic energy (E), the zero-point energy (ZPE) obtained from vibrational frequency analysis was used to correct the electronic energy (E). Thus, the Gibbs free energies for the transformation was calculated as follows: $\Delta G = G_{[Cu12a + 2(PTC4A)]} - G_{[Cu12b + 2(TC4A)]} = E_{[Cu12a + 2(PTC4A)]} - E_{[Cu12b + 2(TC4A)]} + \Delta ZPE$.

Species	E_{ele}	ZPE
[Cu12a + 2(PTC4A)]	-19235.570803 Ha	3.563644 Ha
[Cu12b + 2(TC4A)]	-19235.590445 Ha	3.571621 Ha

1 Ha = 627.5094 kcal/mol.

X. Synthesis of Cbz-PrAH (9-(prop-2-yn-1-yl)-9H-carbazole)

Cbz-PrAH was prepared by referring the reported procedures.[8] Carbazole (1.00 g, 5.98 mmol) was dissolved in 10 mL acetone, then KOH (0.50 g, 8.97 mmol) was added successively. The reaction was stirred at 65°C for 2 h, then 3-bromopropyne (1.42 g,

11.9 mmol) was added successively and refluxed for 6 h. The solvents were evaporated to obtain white powder (1.07 g, yield: 84%). ^{1}H NMR (400 MHz, DMSO-d_6) δ 8.16 (d, J = 7.7 Hz, 2H), 7.69 (d, J = 8.2 Hz, 2H), 7.63–7.40 (m, 2H), 7.41–7.07 (m, 2H), 5.32 (s, J = 2.4 Hz, 2H), 3.26 (s, J = 2.4 Hz, 1H). ^{13}C NMR (101 MHz, DMSO-d_6) δ 140.09 (s), 126.33 (s), 122.96 (s), 120.83 (s), 119.86 (s), 110.03 (s), 79.66 (s), 74.93 (s), 32.31 (s). ESI-MS *m/z*: Sim. = 206.0964 Exp. = 206.1079 ($[M+H]^+$). (Supplementary Fig. 19)

XI. Synthesis of H_4TC4A (*p-tert*-butylthiacalix[4]arene) and H_4PTC4A (*p-phenyl*-thiacalix[4]arene)

H_4TC4A and H_4PTC4A were prepared by referring the reported procedures.[9]

H_4TC4A: A mixture of *p-tert*-butylphenol (32.3 g, 0.22 mol), elemental sulfur S_8 (13.8 g, 0.43 mol), and NaOH (4.43 g, 0.107 mol) in tetraethylene glycol dimethyl ether (10 cm^3) was stirred under nitrogen. The stirred mixture was heated gradually to 230ºC over a period of 5 h and kept at this temperature for further 4 h. The resulting dark red product was cooled to ambient temperature and diluted with toluene (20 cm^3) and 2 M aq. sulfuric acid solution (70 cm^3), followed by addition of diethyl ether (80 cm^3) with stirring to give a suspension. The precipitate was collected by filtration, recrystallized from chloroform and dried in vacuo (100ºC, 4 h) to give a pure sample of H_4TC4A (18.9 g, yield: 49%). ^{1}H NMR (400 MHz, $CDCl_3$) δ 9.59 (s, 4H), 7.63 (s, 8H), 1.21 (s, 36H). ^{13}C NMR (101 MHz, $CDCl_3$) δ 155.65 (s), 144.71 (s), 136.42 (s), 120.56 (s), 34.23 (s), 31.27 (s). ESI-MS *m/z*: Sim. = 719.2352 Exp. = 719.2509 ($[M-H]^-$). (Supplementary Fig. 20)

H_4PTC4A: The synthesis of H_4PTC4A was similar to that described for H_4TC4A, except the *p-tert*-butylphenol was replaced with *p*-phenylphenol. ^{1}H NMR (400 MHz, $CDCl_3$) δ 9.59 (s, 4H), 7.81 (s, 8H), 7.38–7.21 (m, 20H). ^{13}C NMR (101 MHz, $CDCl_3$) δ 156.21 (s), 137.92 (s), 136.91 (s), 134.43 (s), 127.88 (s), 126.46 (s), 125.78 (s), 120.16 (s). ESI-MS *m/z*: Sim. = 799.1100 Exp. = 799.2068 ($[M-H]^-$). (Supplementary Fig. 21)

XII. Synthesis of Cbz-PrACu precursor.

$CuSO_4·5H_2O$ (5.00 g, 20.0 mmol) was dissolved at a mixed solution of 20 mL $NH_3·H_2O$ and 80 mL H_2O, then $NH_2OH·HCl$ (1.39 g, 20.0 mmol) was added successively. During this period the color of the solution changes from dark blue to a

colorless, then Cbz-PrAH (4.11 g, 20.0 mmol) was dissolved at 10 mL acetone and added successively. The resulting mixture was stirred at 25°C for 12 h. Filtration yields material that is washed twice with 50 mL water, once with 50 mL ethanol, once with 10 mL diethyl ether to yield 5.01 g (93.4 %) of product. Selected IR peaks (cm^{-1}): 3300 (w), 3044 (w), 1589 (m), 1460 (s), 1329 (s), 1214 (s), 1152 (m), 1066 (m), 923 (s), 747 (s), 717 (s). Elemental analyses calc. for $C_{15}H_{10}NCu$: C, 67.28; H, 3.76; N, 5.23; found: C, 67.36; H, 3.76; N, 5.29. (Supplementary Fig. 22-23)

XIII. TD-DFT calculation of Cu12a and Cu12b.

To understand the origin of the optical absorption of **Cu12a** and **Cu12b**, TD-DFT calculation via Gaussian 16 were performed. It was apparent that both **Cu12a** and **Cu12b** featured similar electronic structures (Supplementary Fig.24). The 3d states of Cu atoms and 2p sates of C, S, O and N atoms play a crucial role in the conduction band minimum (CBM), while only 3d4s states of all Cu atoms and 2p states of C atoms dominated in the valence band maximum (VBM). Therefore, we tentatively inferred that the origin of the optical absorption might be attributed to electronic transitions from the peripheral ligands to the inner Cu core and metal-perturbed ligand-to ligand. In order to further gain access to the electronic structure and deeply understand the underlying reason of the variant absorbance ratios of absorption peaks between two Cu_{12} clusters, absorption band composition analyses were studied (Supplementary Fig. 25-27 and Supplementary Table 5-6). The excitation of lower energies (362.36 nm for **Cu12a** and 337.40 nm for **Cu12b**) in calculated spectra corresponds to the 340 nm peak in experimental data, both primarily arising from $L_{(Cbz\text{-}PrA+TC4A)}M_{(Cu)}CT$ or $L_{(Cbz\text{-}PrA+PTC4A)}M_{(Cu)}CT$ transitions. Nevertheless, the excitation at high energies (297.75 nm for **Cu12a** and 294.16 nm for **Cu12b**) show different components. The excitation of **Cu12a** at 297.75 nm primarily arise from transition out of the HOMO-1, HOMO-2 and HOMO-5 into the LUMO+6, LUMO+8 and LUMO+11 orbitals. These orbitals are all comprised of the *p* character from the aromatic rings of $TC4A^{4-}$ ligand, atomic *d* character from Cu_{12} core, as well as small amount of *p* character from $Cbz\text{-}PrA^{-}$ ligand, which comprise the $L_{(TC4A)}M_{(Cu)}CT$ and $L_{(TC4A)}L_{(Cbz\text{-}PrA)}CT$ transitions. In contrast, the excitation of **Cu12b** at 294.16 nm arise from HUMO-2, HOMO-7, HOMO-8 and

HOMO-21 dominated by Cbz-PrA$^-$ and PTC4A^{4-} ligands into LUMO+1, LUMO+6, LUMO+7 and LUMO+10 dominated by Cu_{12} core, Cbz-PrA$^-$ and PTC4A^{4-} ligands, which also mainly comprise the $L_{(PTC4A)}M_{(Cu)}CT$ and $L_{(PTC4A)}L_{(Cbz\text{-}PrA)}CT$. These orbitals of **Cu12b** show nearly same character with **Cu12a**, except the extra transition partially locate in π/π* orbitals of Cbz-PrA$^-$ and PTC4A^{4-} ligands, which corresponding to $IL_{(Cbz\text{-}PrA+PTC4A)}CT$ transition. One can thus concluded that the heavy mixing of $IL_{(Cbz\text{-}PrA+PTC4A)}CT$ transition in **Cu12b** makes up the stronger absorption intensity of this peak compared to **Cu12a**, which also explain the absorbance ratios of double absorption peaks of two clusters are significantly different.

XIV. Recyclable performance experiments.

The iodine loaded porous supramolecular framework (PSF) **I_2@Cu12a-π** (5 mg) was soaked in several solvents (ethanol: EtOH, acetonitrile: CH_3CN and ethyl acetate: EA) for about 30 minutes to release the absorbed iodine. In which, the absorbed iodine can be effectively released in ethyl acetate, and the recovered dry PSF powder was reused for additional iodine adsorption tests.

XV. Mass spectroscopy.

Electrospray ionization mass spectra (ESI-MS) were recorded on a Bruker impact II high definition mass spectrometer, quadrupole and time-of-flight (Q/TOF) modules in the positive-ion mode. Typical measurement conditions are as follows: end plate offset = 500 V; dry gas = 4 L/min, nebulizer = 0.3 bar, capillary voltage = 4500 V, sample flow rate = 4 μL/min. The data analyses of mass spectra were performed based on the isotope distribution patterns using Agilent MassHunter Workstation Data acquisition software (Version B.05.00). The reported *m/z* values represent monoisotopic mass of the most abundant peak within the isotope pattern.

XVI. Elemental analysis.

The elemental analyses (C, H, and N) were determined on a Vario EL III analyzer.

Section 2. Supplementary Figures and Tables

Supplementary Fig. 1 Synthesis routes for Cbz-PrACu and Cu12a. The **Cu12a** was formed by solvothermal reaction. MeOH = methanol, EA = ethyl acetate.

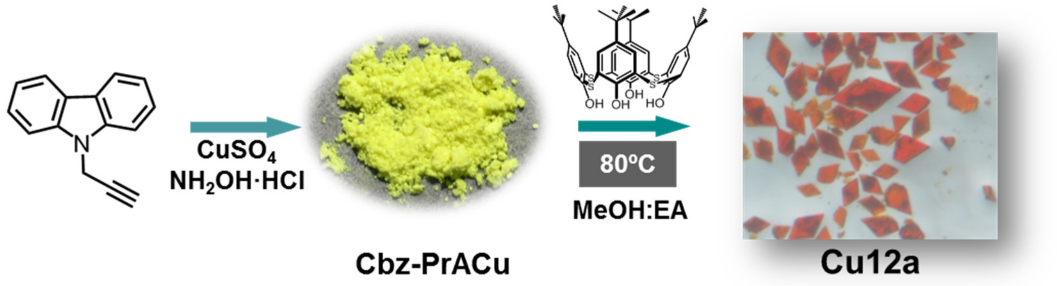

Supplementary Fig. 2 SEM and elemental mapping images of Cu12a (a) and Cu12b (b).

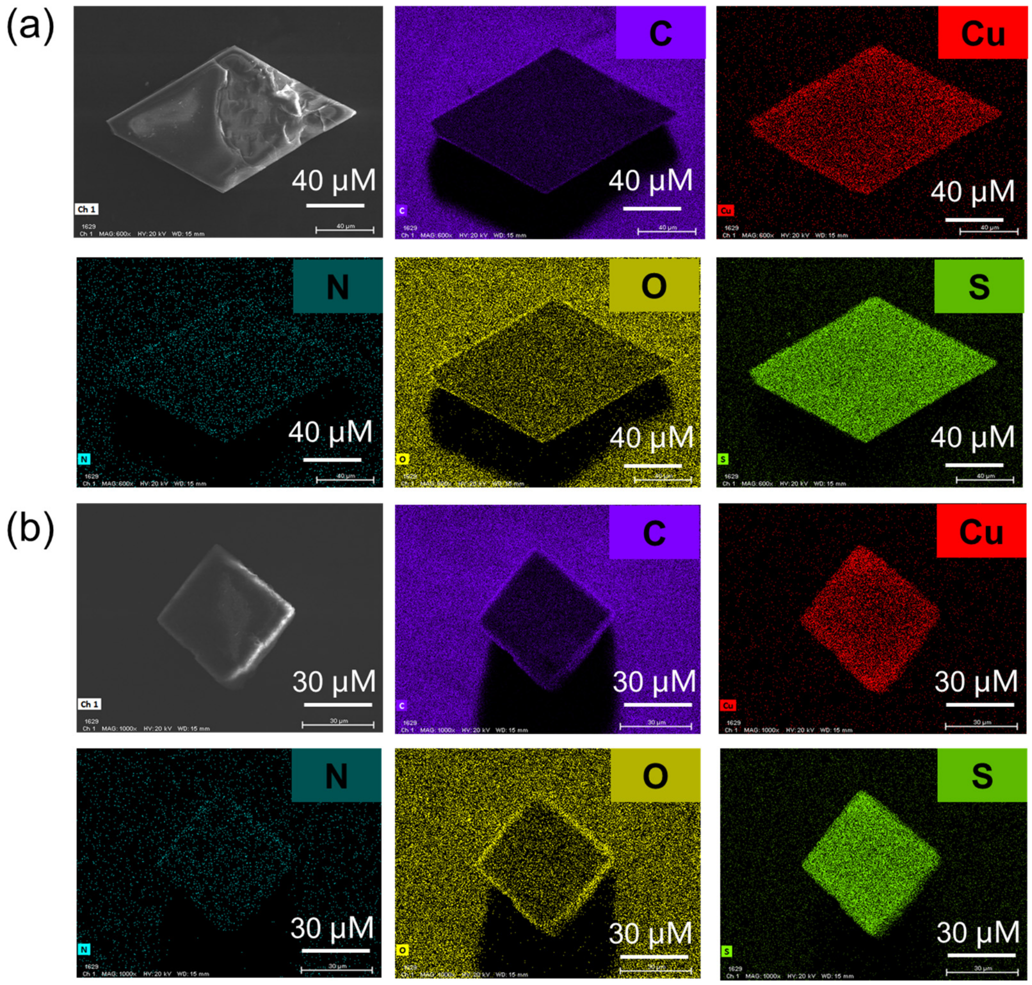

Supplementary Fig. 3 The infrared (IR) spectra of Cu12a (a) and Cu12b (b).

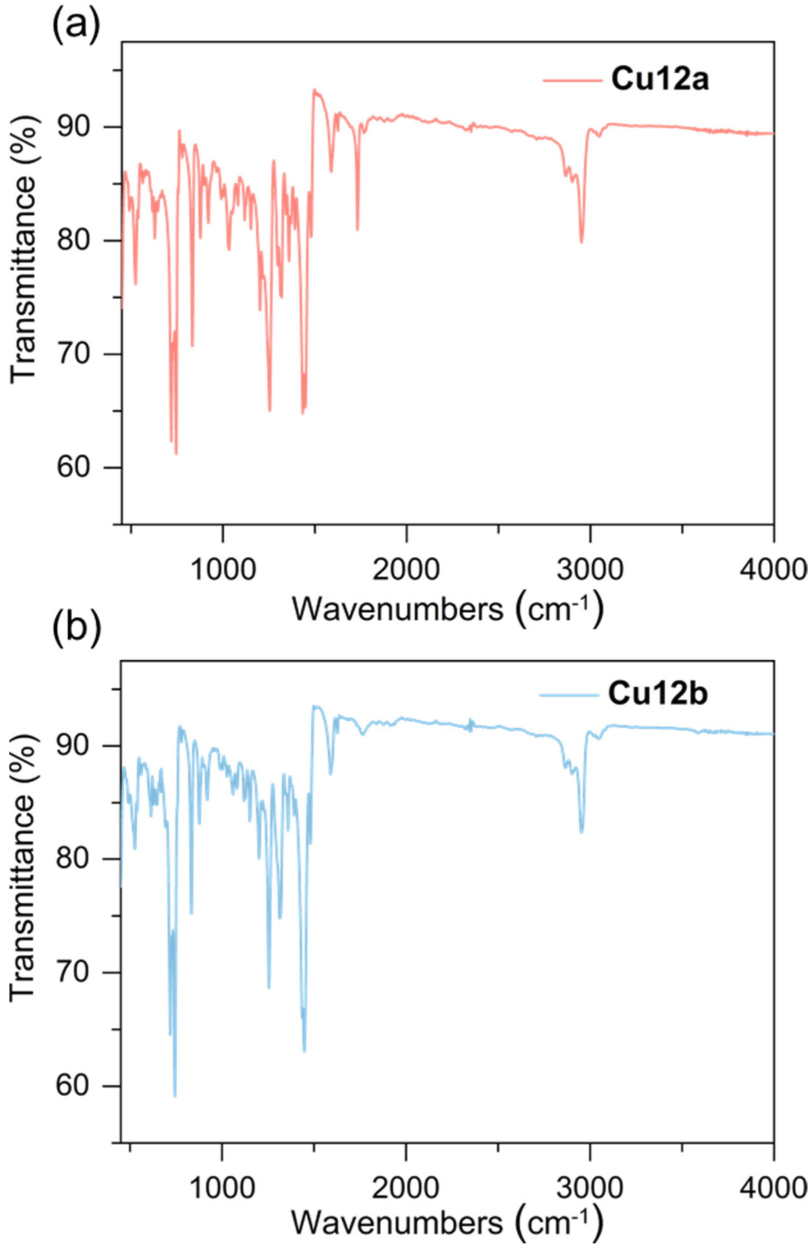

Supplementary Fig. 4 The surfaces of Cu12a (a) and Cu12b (b) calculated via 3V Volume Assessor program[10] by rolling a virtual probe (1.0 Å) on the surface viewed along six different orientations.

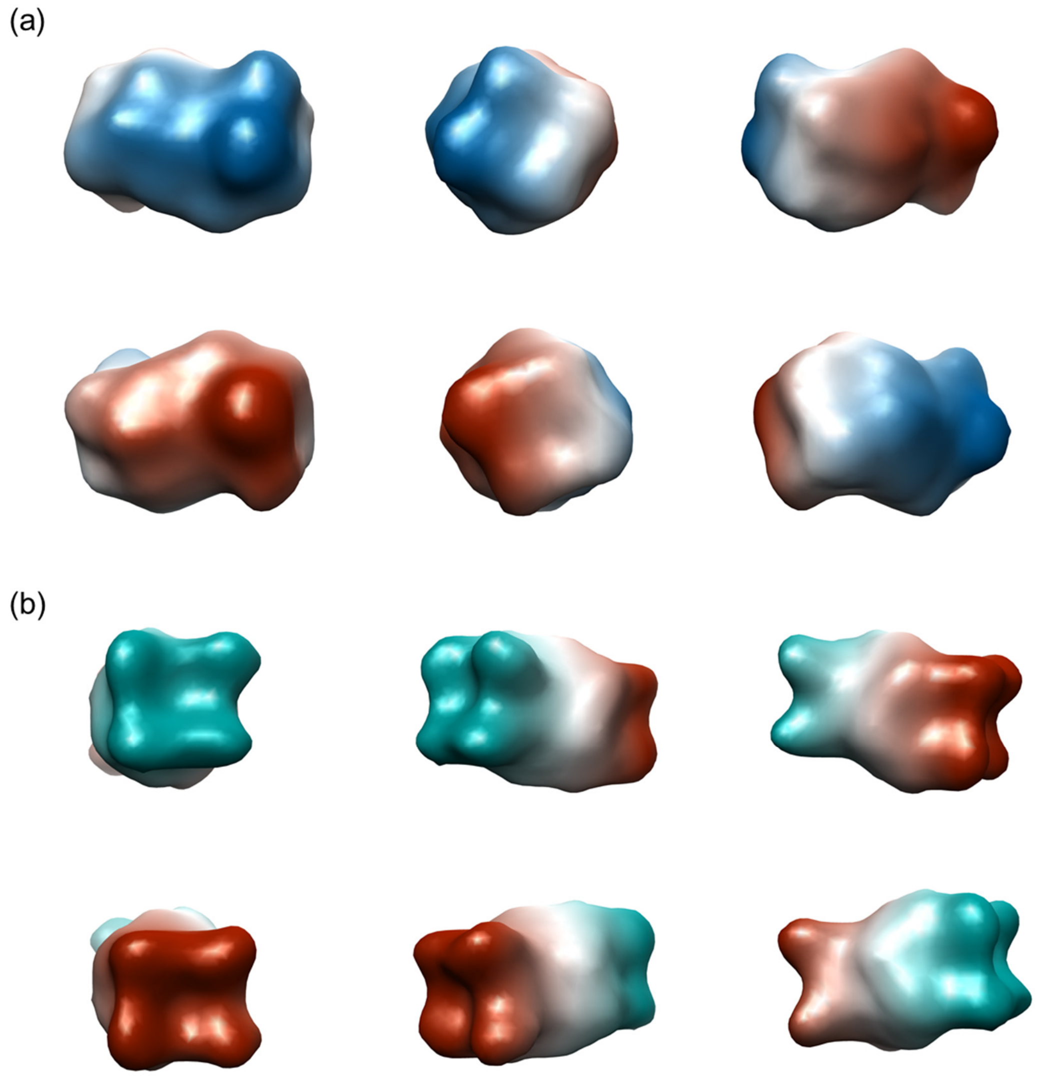

Supplementary Fig. 5 The asymmetric units of Cu12a (a) and Cu12b (b). Color legend: purple, Cu; gray, C; red, O; blue, N; yellow, S.

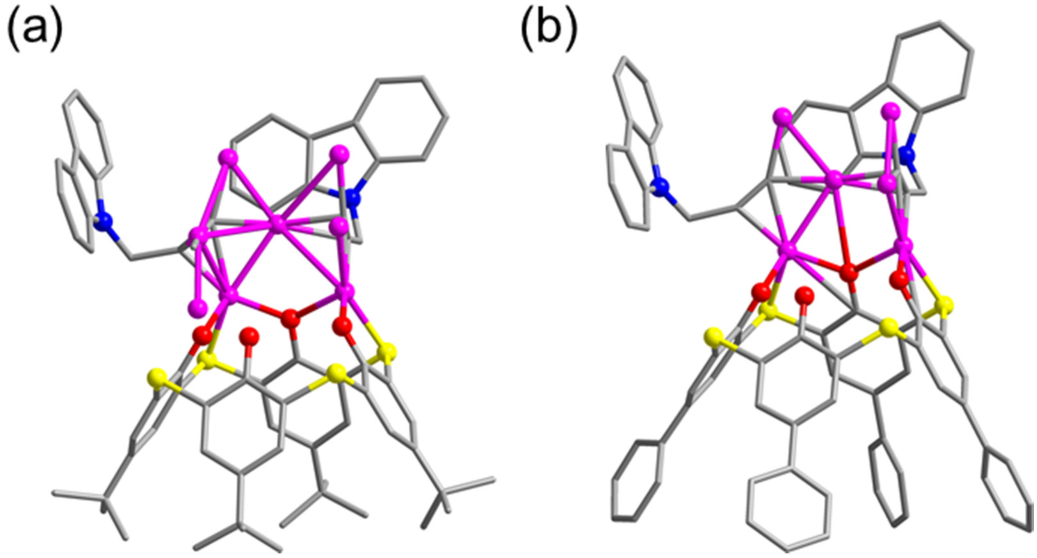

Supplementary Fig. 6 Four Cbz-PrA⁻ ligands coordinated with Cu_{12} kernel in Cu12a and Cu12b (a). Coordination mode of Cbz-PrA⁻ (b). Coordination mode of $TC4A^{4-}$ (c) and $PTC4A^{4-}$ ligands (d). Color legend: purple, Cu; gray, C; blue, N; yellow, S; red, O.

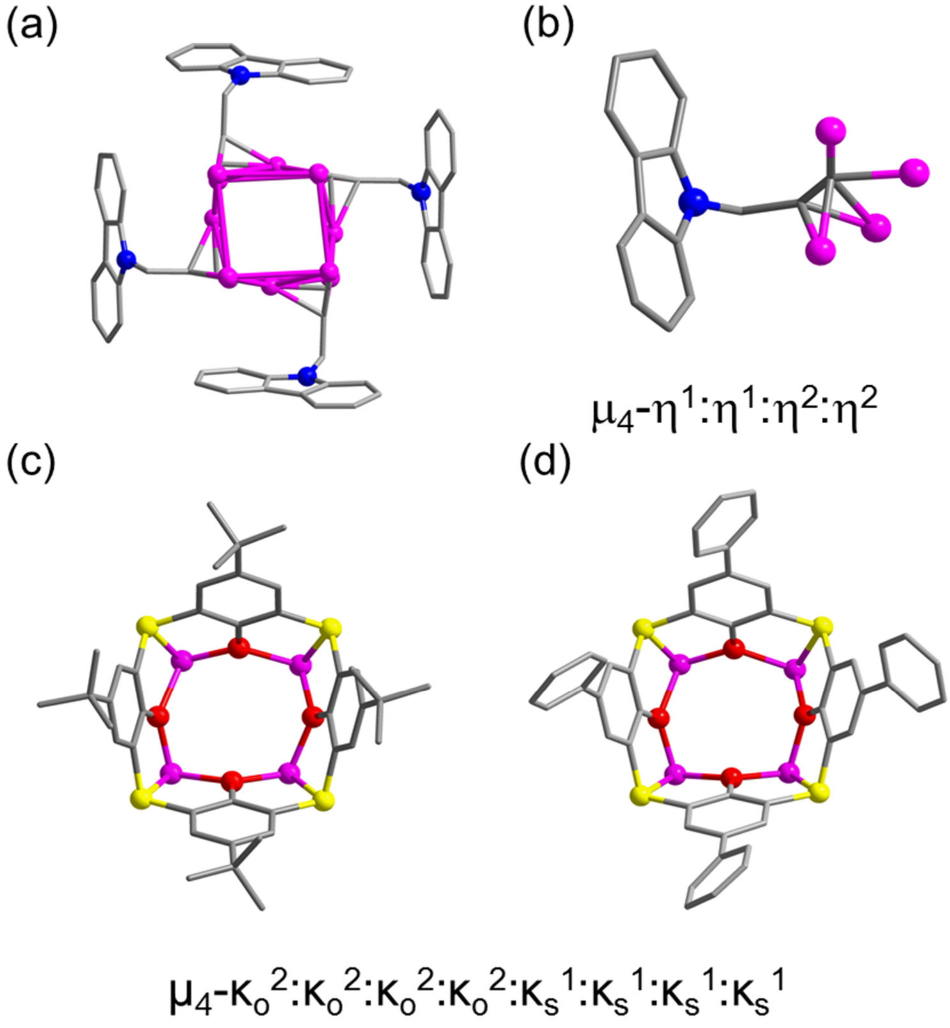

Supplementary Fig. 7 The molecular packing arrangements of Cu12a (a and b), Cu12a-no solvent (c and d) and Cu12a-π (e and f) along the *b* and *c* axes.

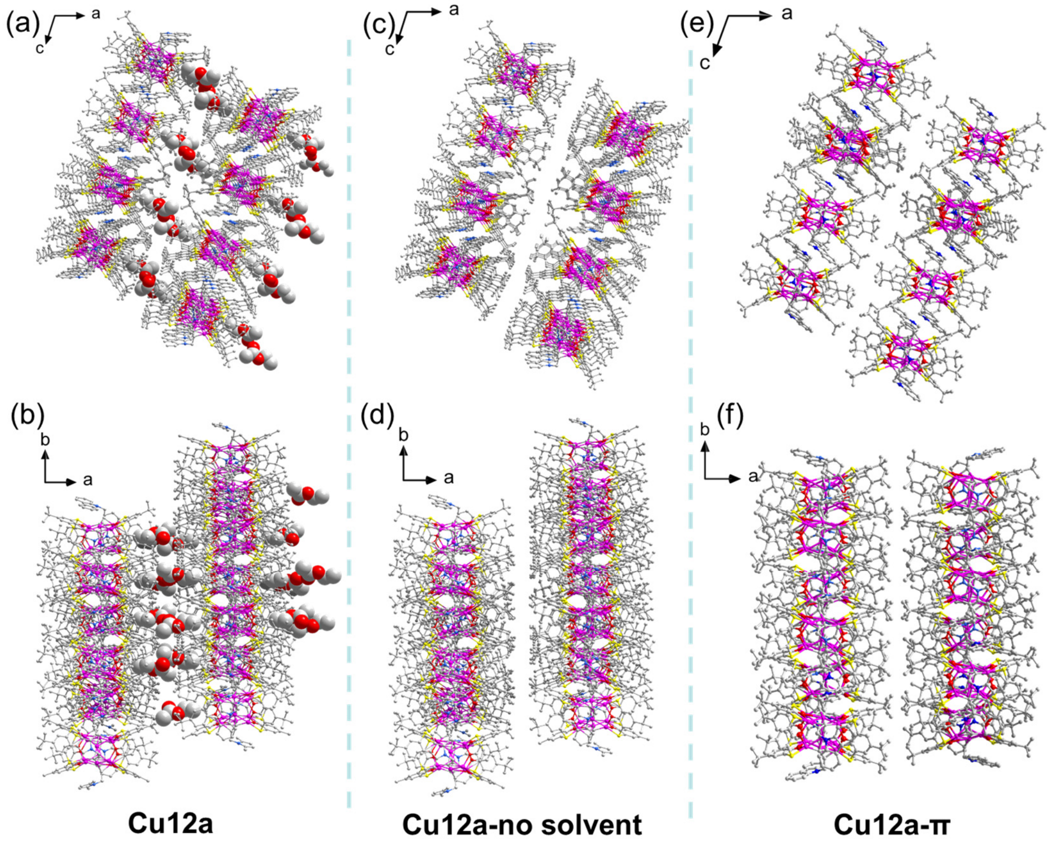

Supplementary Fig. 8 (a) The Cu_{12} cluster in Cu12a displaying a trumpet-type motif. (b) The intralayer C-H···π interactions between Cu_{12} clusters. The top (c) and side (d) views of supramolecular framework of As-prepared PSF.

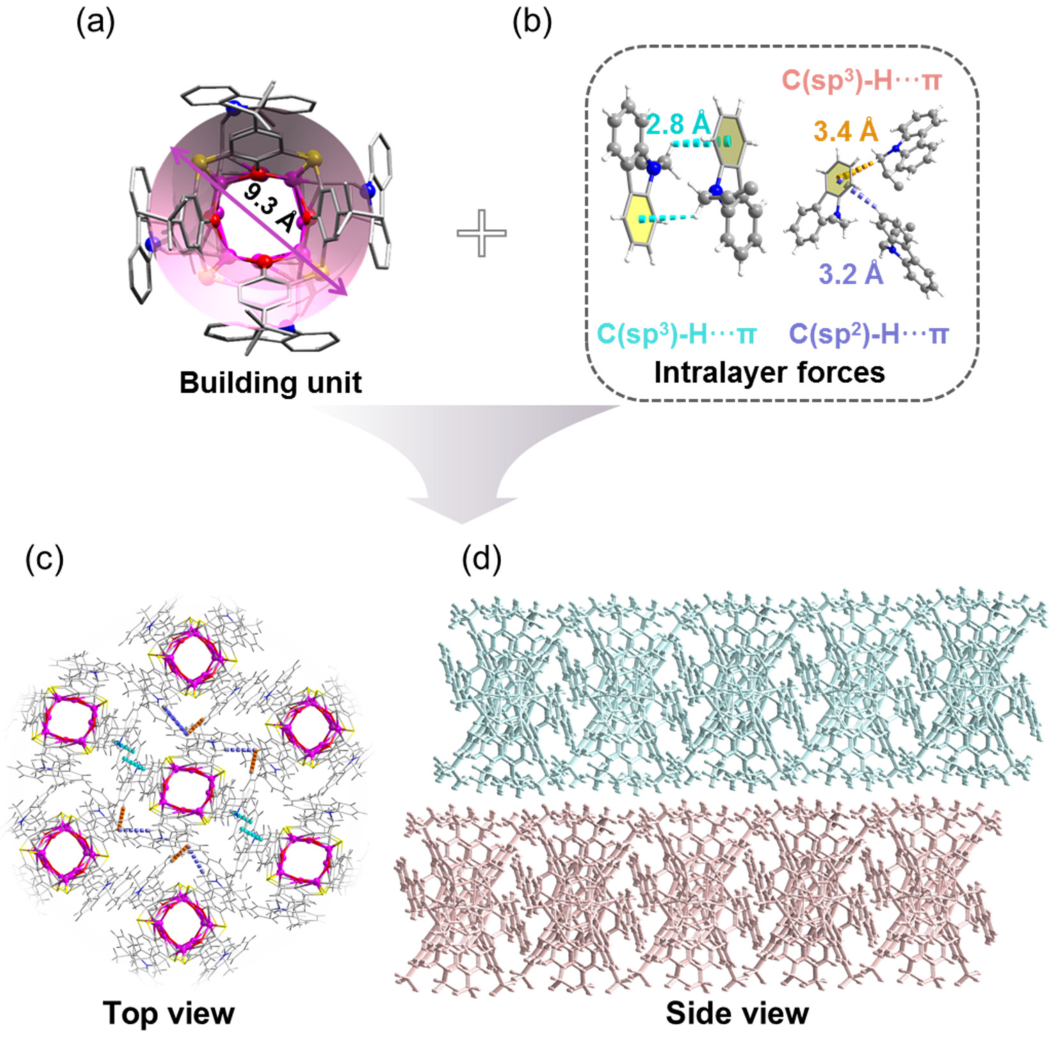

Supplementary Fig. 9 (a) BET area calculation for Cu12a-π that fulfils all consistency criteria. Points are selected based on the first consistency criterion. (b) Langmuir surface area plot for Cu12a-π. (c) The pore size distribution of Cu12a-π.

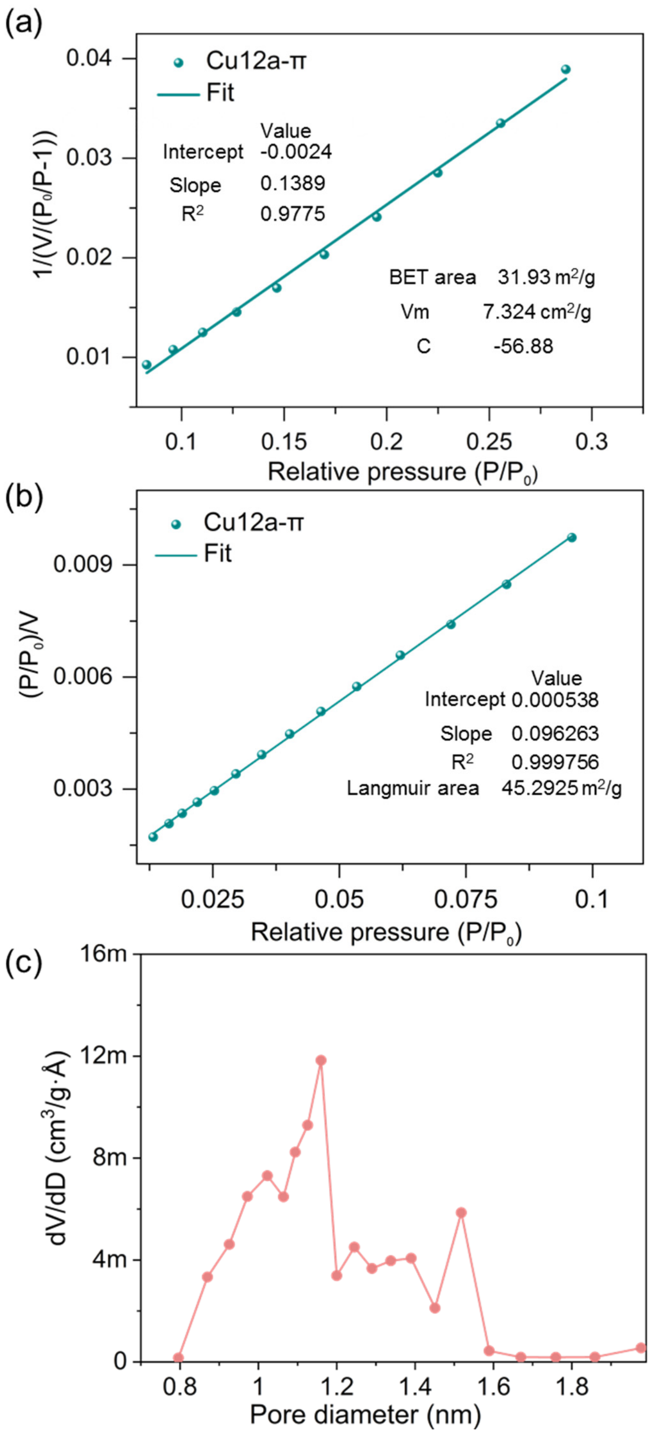

Supplementary Fig. 10 (a) UV-Vis absorption spectra of Cu12a and Cu12b in dichloromethane. (b) The photograh of reaction solution over the time course with the addition of H_4PTC4A. (c) The ratio of $A_{300\ nm}/A_{340\ nm}$ over the time course. (d) UV-Vis absorption spectra of H_4PTC4A and H_4TC4A in dichloromethane (20 μM).

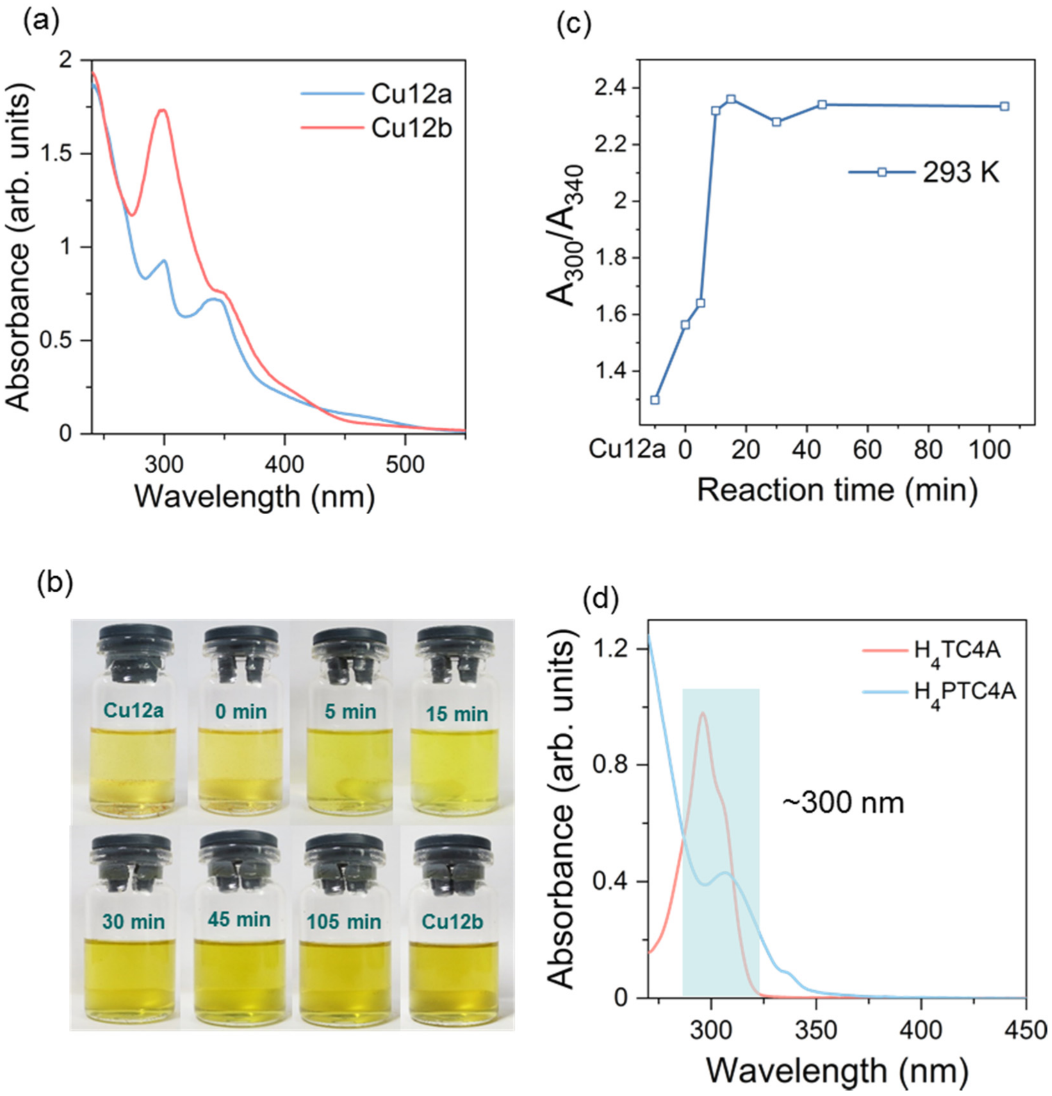

Supplementary Fig. 11 The relative energy difference between the initial and final stages of the transformation reaction calculated by Gaussian 16 (PBE1PBE level).

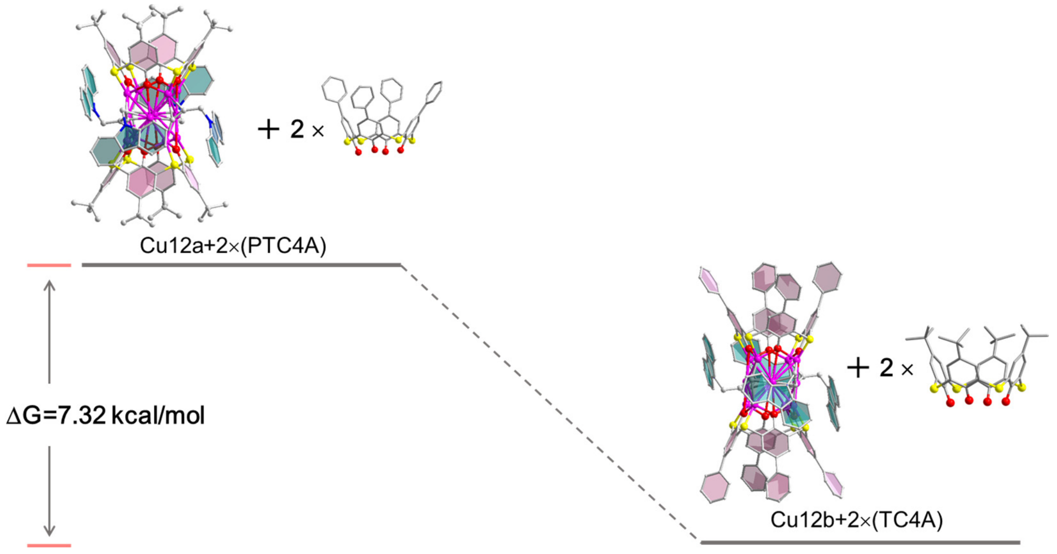

Supplementary Fig. 12 The molecular packing arrangements of Cu12b (a, b and c), Cu12b-no solvent (d, e and f) and Cu12b-NACs (g, h and i) along the *a*, *b* and *c* axes.

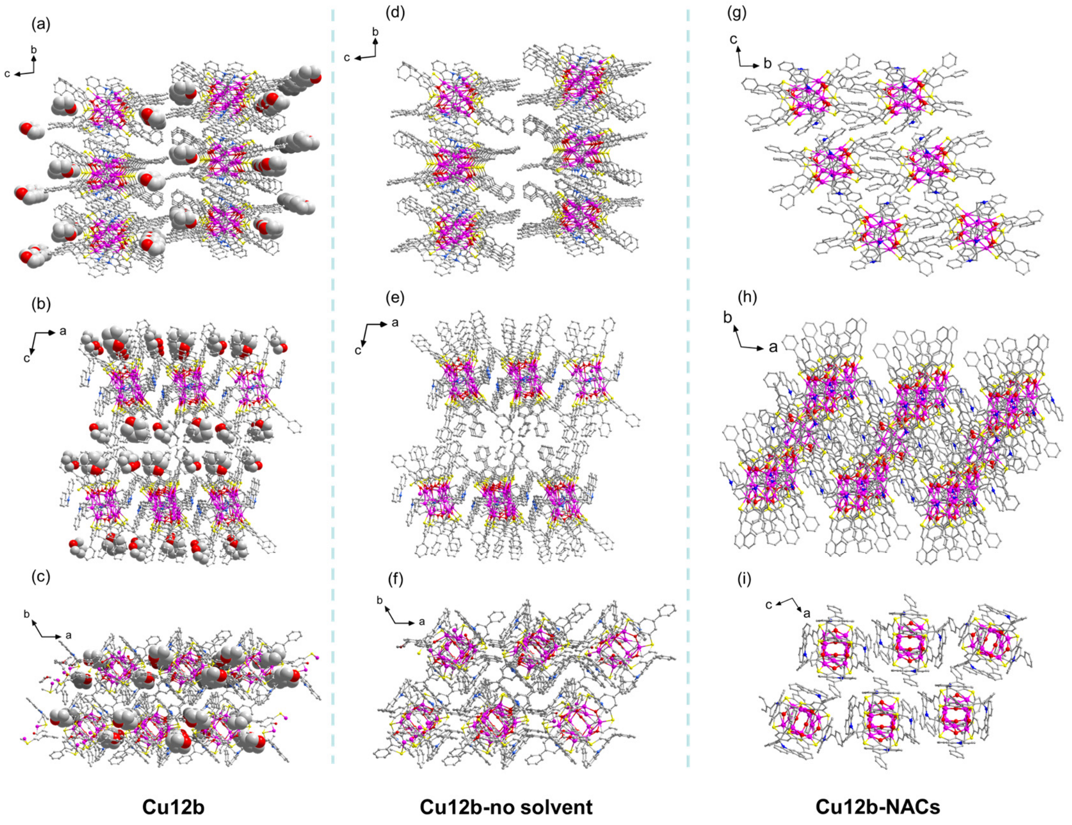

Supplementary Fig. 13 The adjacent Cu_{12} nanoclusters in Cu12b (a) and Cu12b-NACs (b) along the *a* axis.

(a)

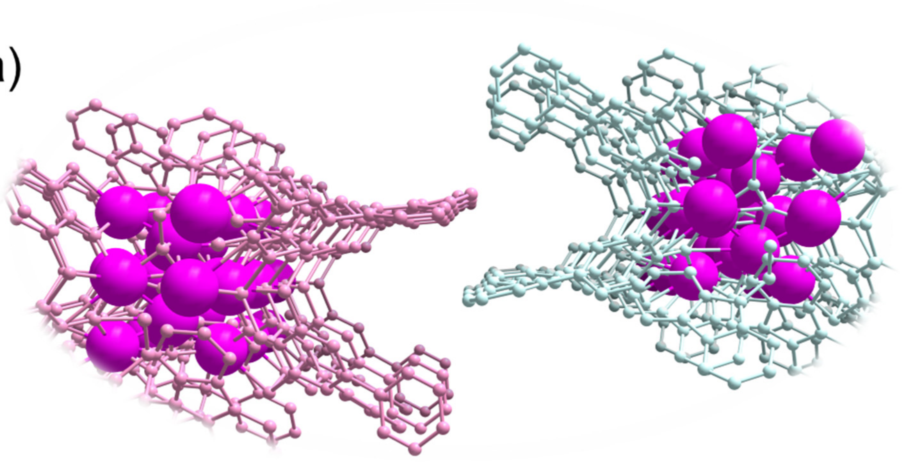

The adjacent Cu_{12} nanoclusters in **Cu12b**

(b)

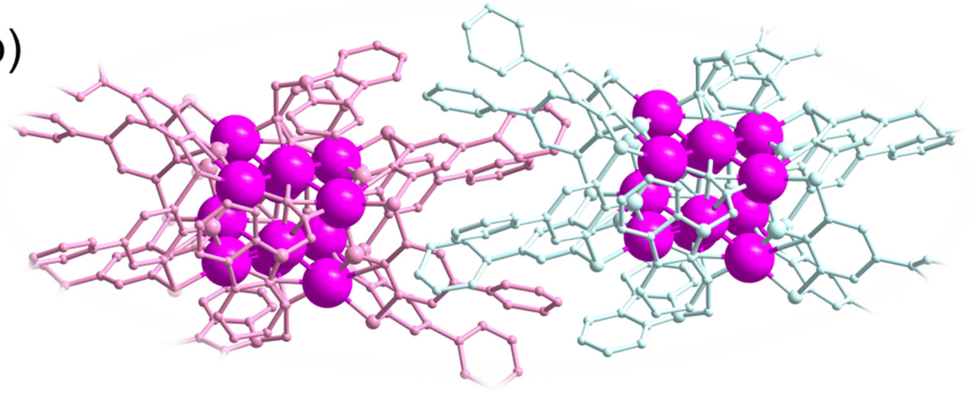

The adjacent Cu_{12} nanoclusters in **Cu12b-NACs**

Supplementary Fig. 14 (a) N_2 adsorption and desorption isotherms of Cu12b-NACs at 77 K (left); CH_4, CO_2, C_2H_4 and C_2H_6 adsorption isotherms of Cu12b-NACs at 273 K (right). (b) Pore size distribution analysis based on Hovath–Kawazoe model. (c) BET area calculation for Cu12b-NACs that fulfils all consistency criteria. Points are selected based on the first consistency criterion. (d) Langmuir surface area plot for Cu12b-NACs.

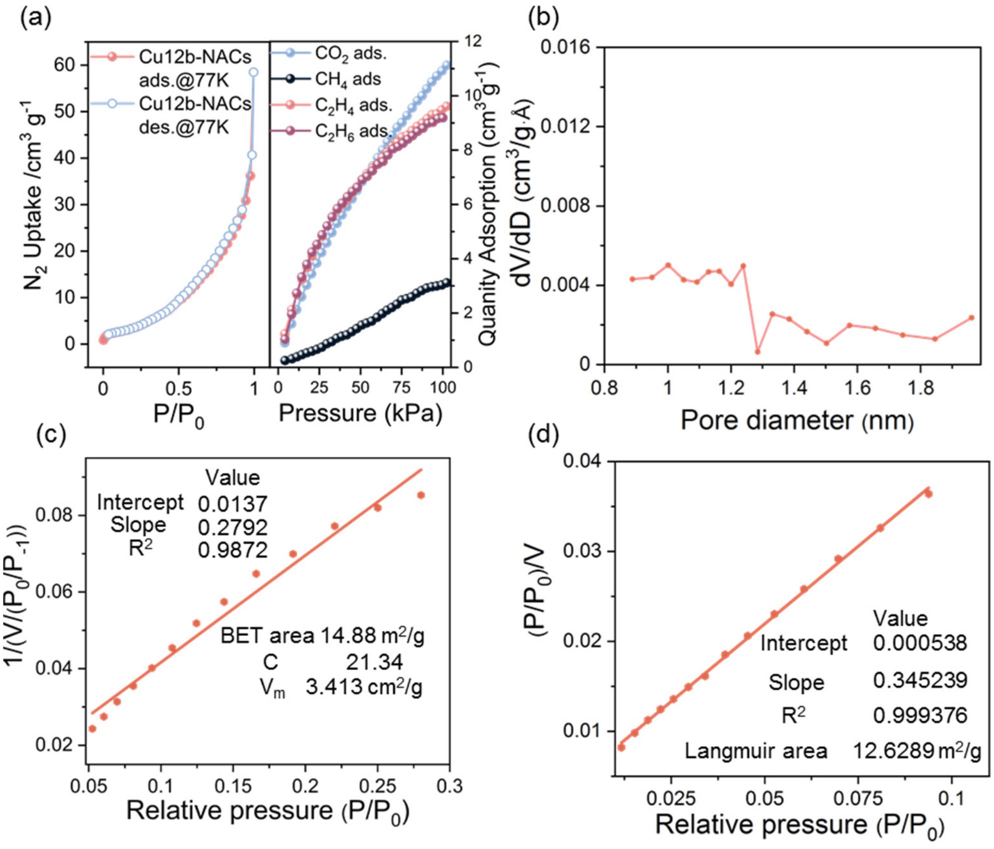

Supplementary Fig. 15 (a) UV-Vis spectra of iodine aqueous solutions at different concentrations. (b) Standard curve plotted based on the absorbance at 461 nm.

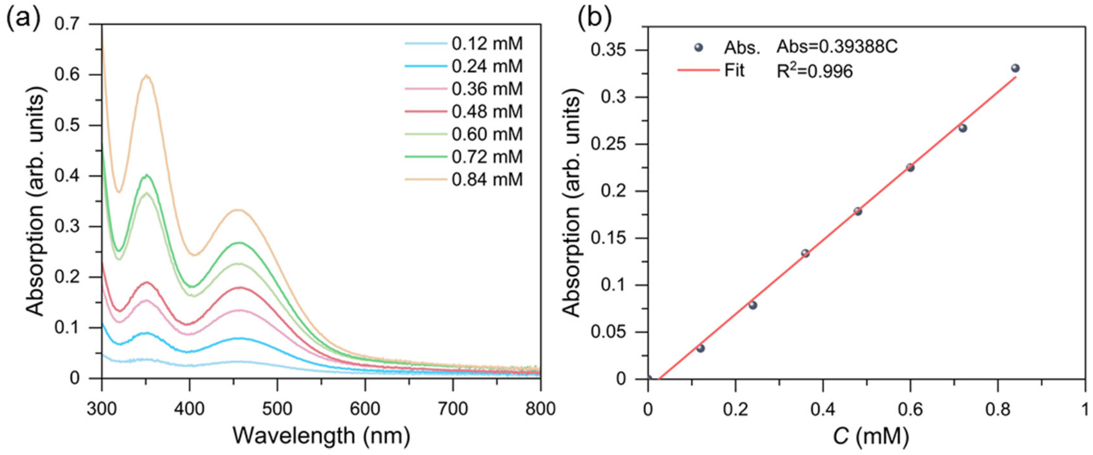

Supplementary Fig. 16 (a) Color changes of saturated iodine aqueous solutions upon addition of Cu12a-π. (b) Time-dependent UV-Vis adsorption spectra of saturated iodine aqueous solution (1.2 mM) recorded after contacting with Cu12a-π (0.1 mg mL^{-1}).

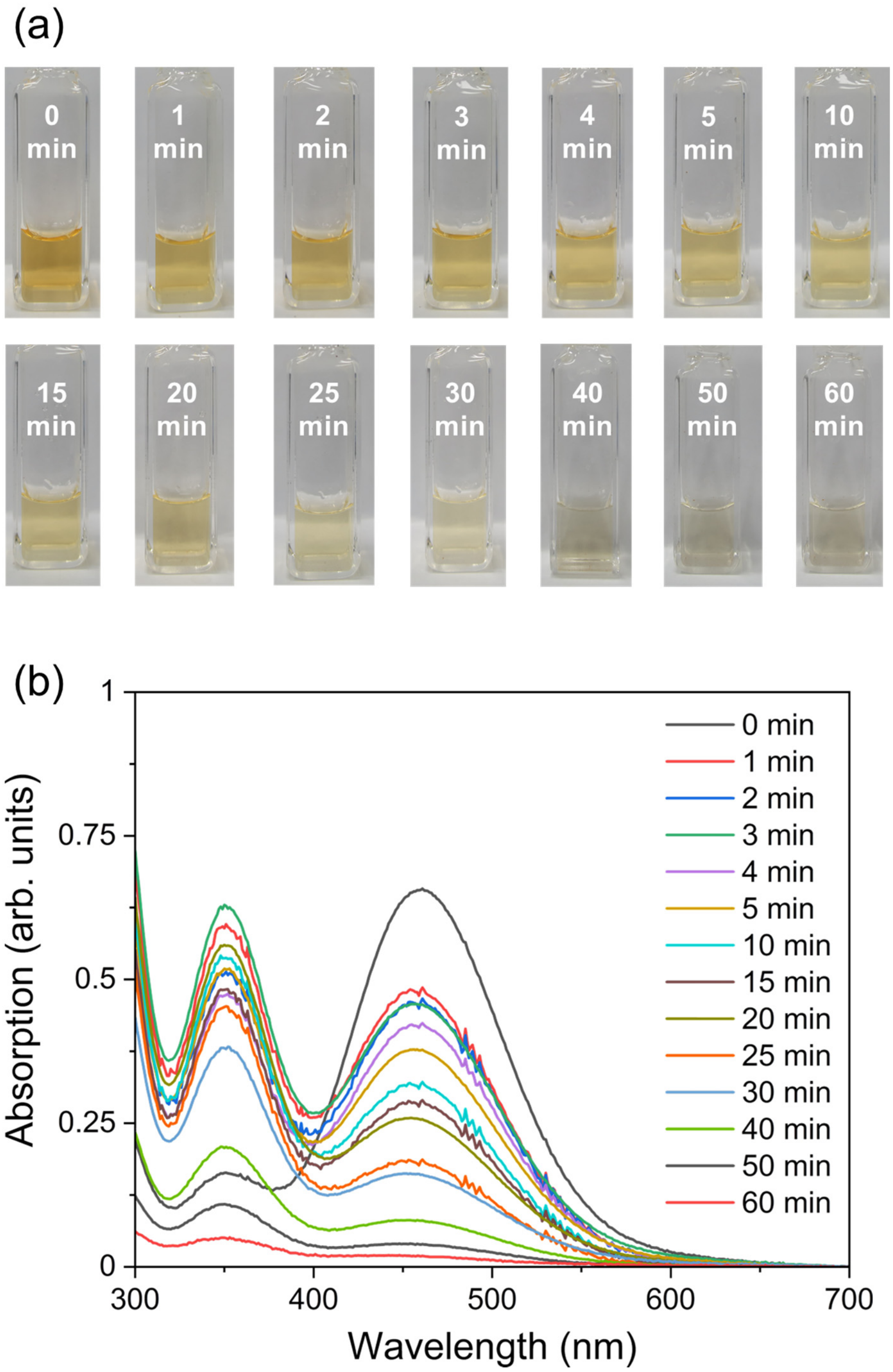

Supplementary Fig. 17 (a) Color changes of saturated iodine aqueous solutions upon addition of Cu12b-NACs (b) Time-dependent UV-Vis adsorption spectra of saturated iodine aqueous solution (1.2 mM) recorded after contacting with Cu12b-NACs (0.1 mg mL^{-1}).

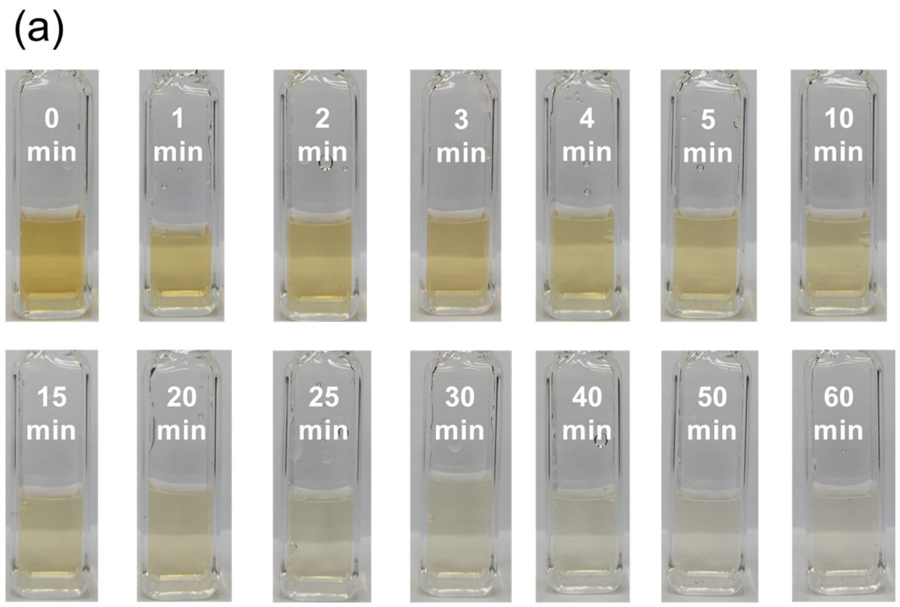

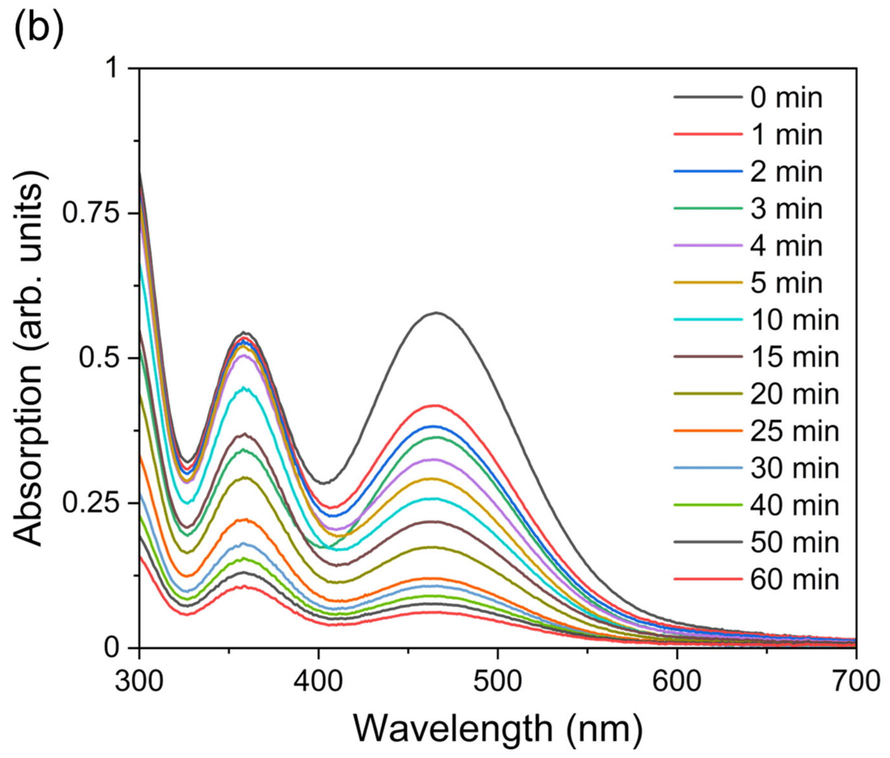

Supplementary Fig. 18 Color changes of I_2@Cu12a-*π* soaked in several solvents (EtOH, CH_3CN and EA) for 30 minutes.

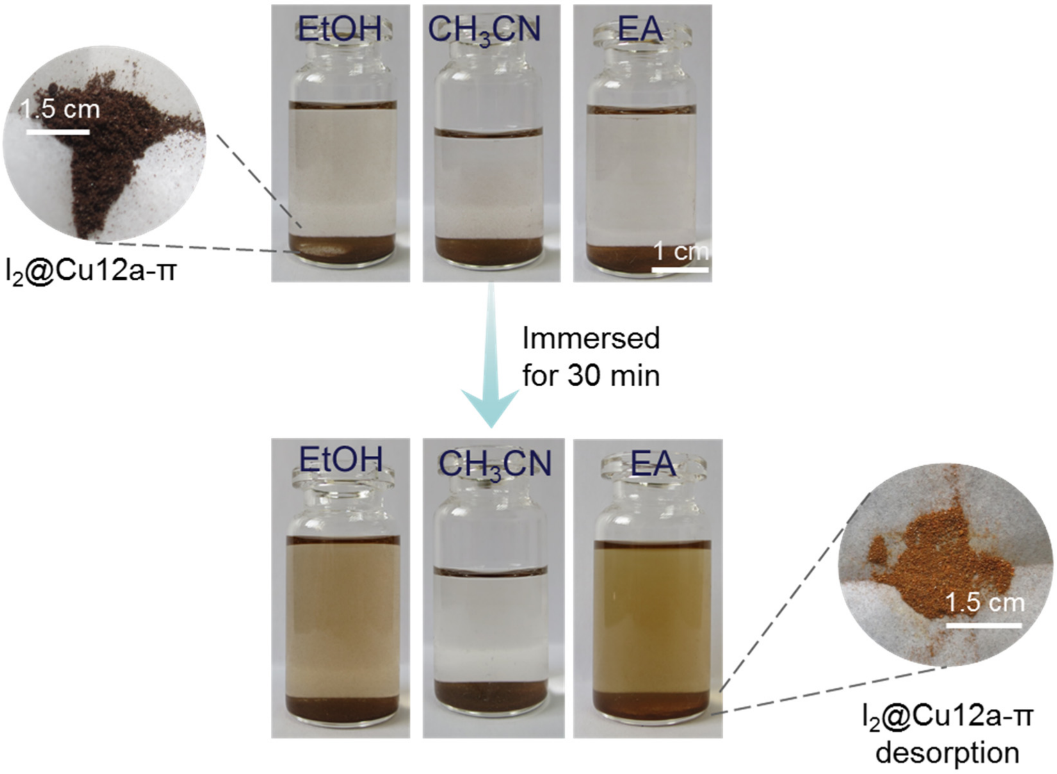

Supplementary Fig. 19 ^{1}H NMR, ^{13}C NMR and mass spectra of Cbz-PrAH.

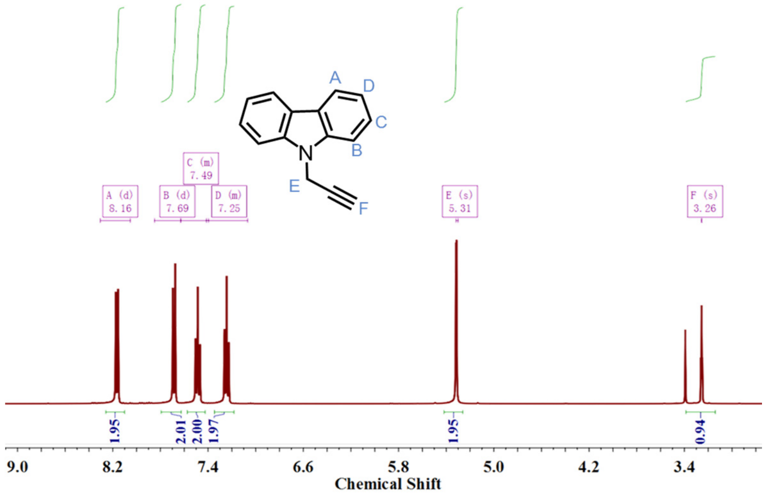

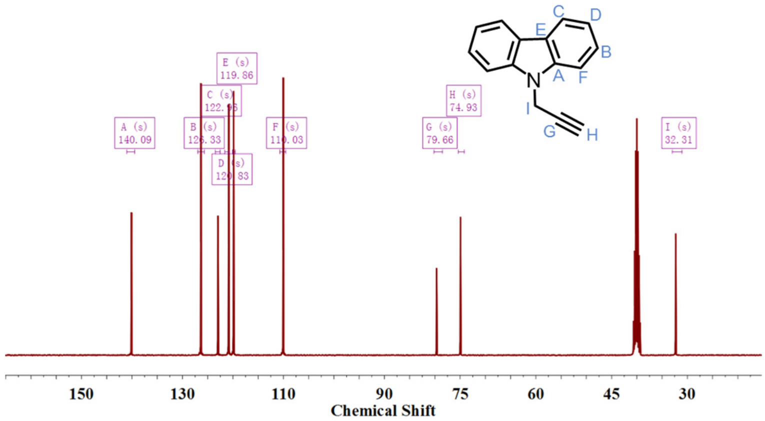

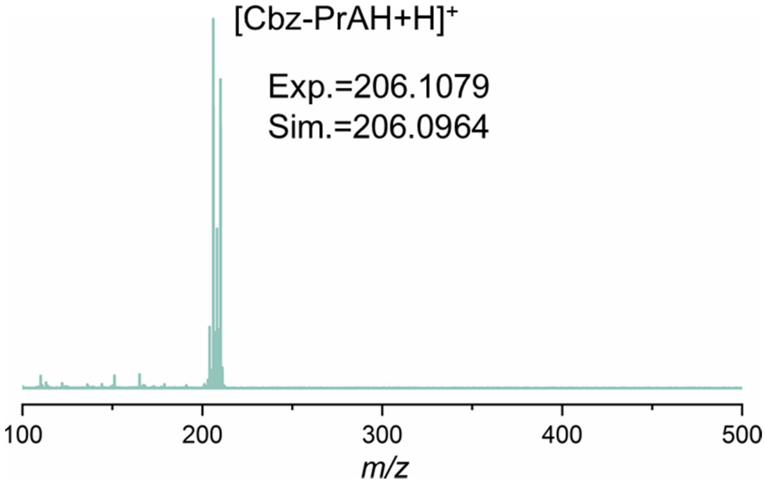

Supplementary Fig. 20 ^{1}H NMR, ^{13}C NMR and mass spectra of H_4TC4A.

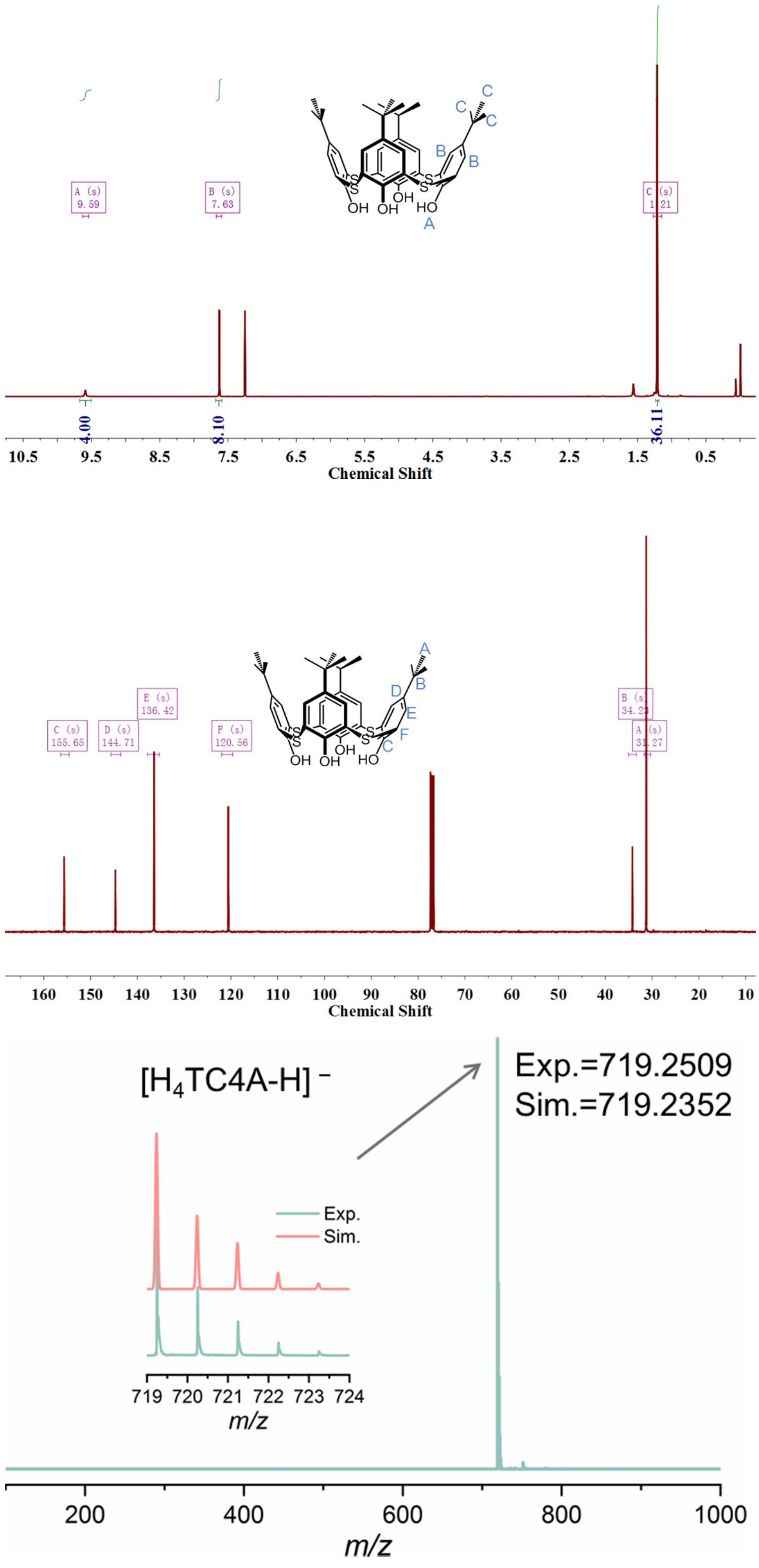

Supplementary Fig. 21 ^{1}H NMR, ^{13}C NMR and mass spectra of H_4PTC4A.

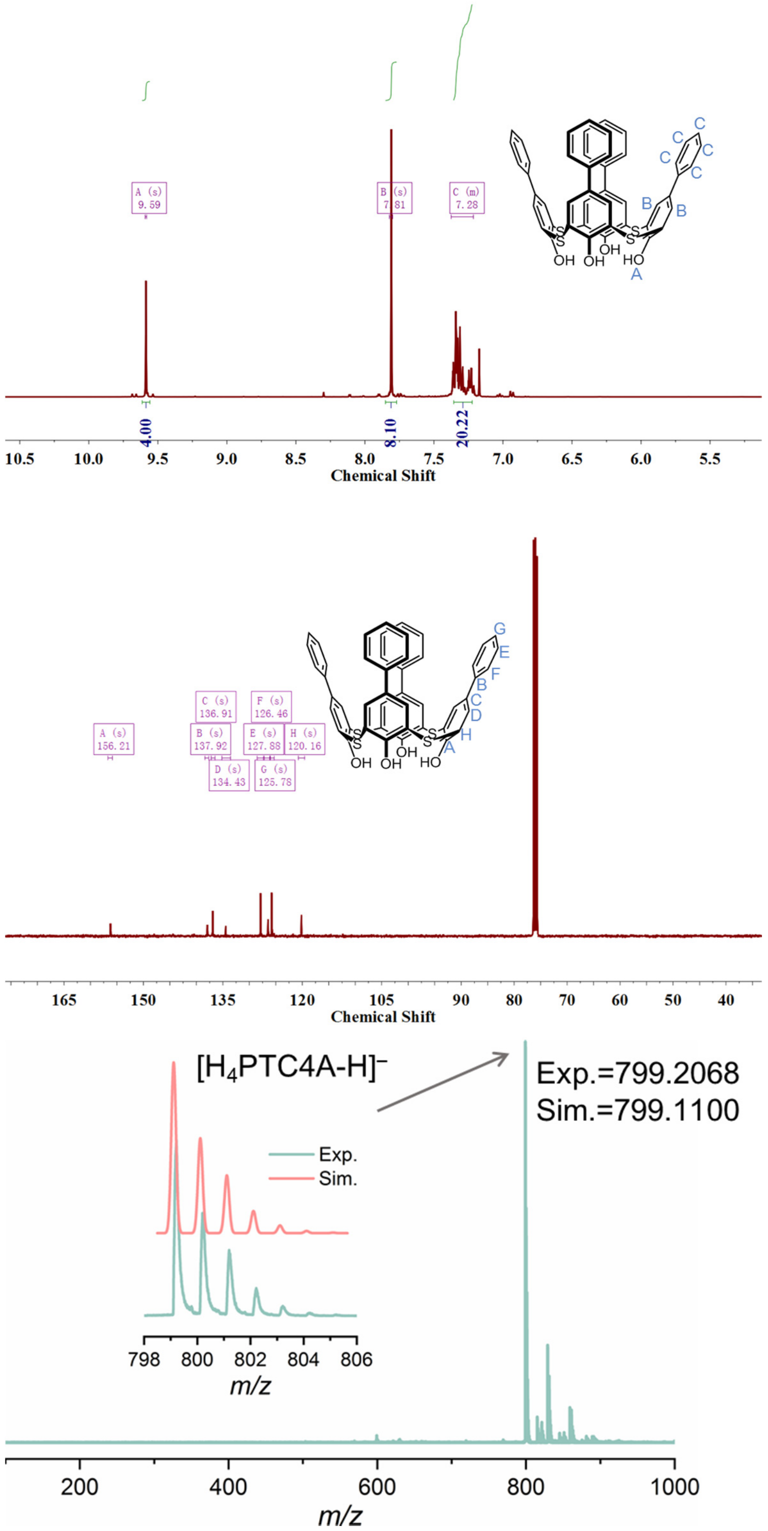

Supplementary Fig. 22 The infrared (IR) spectrum of Cbz-PrACu precursor.

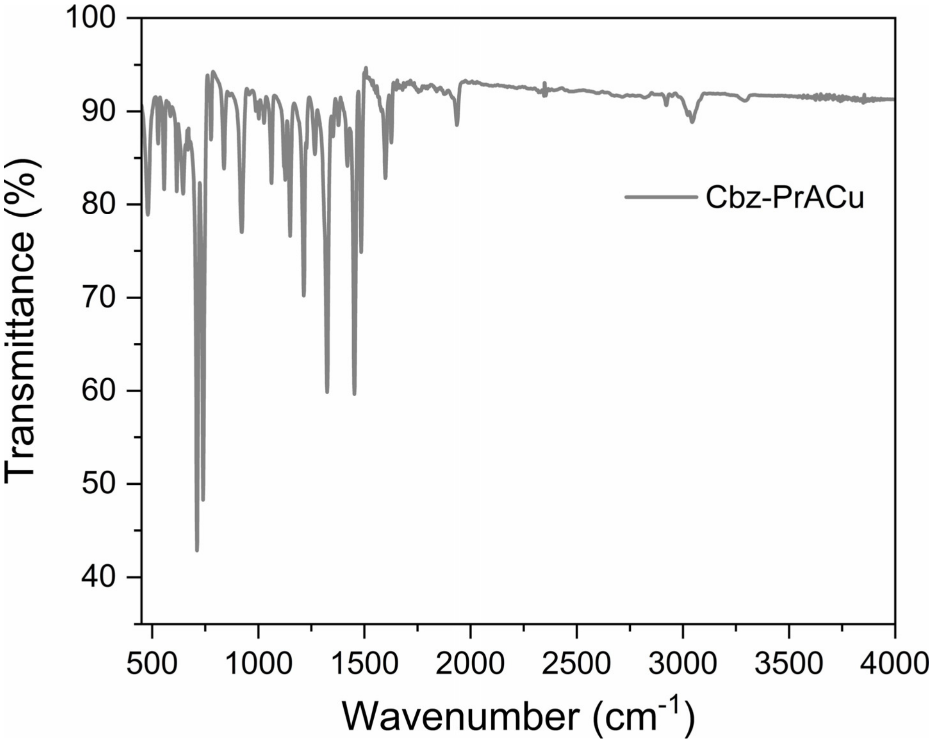

Supplementary Fig. 23 TGA curve of Cbz-PrACu precursor.

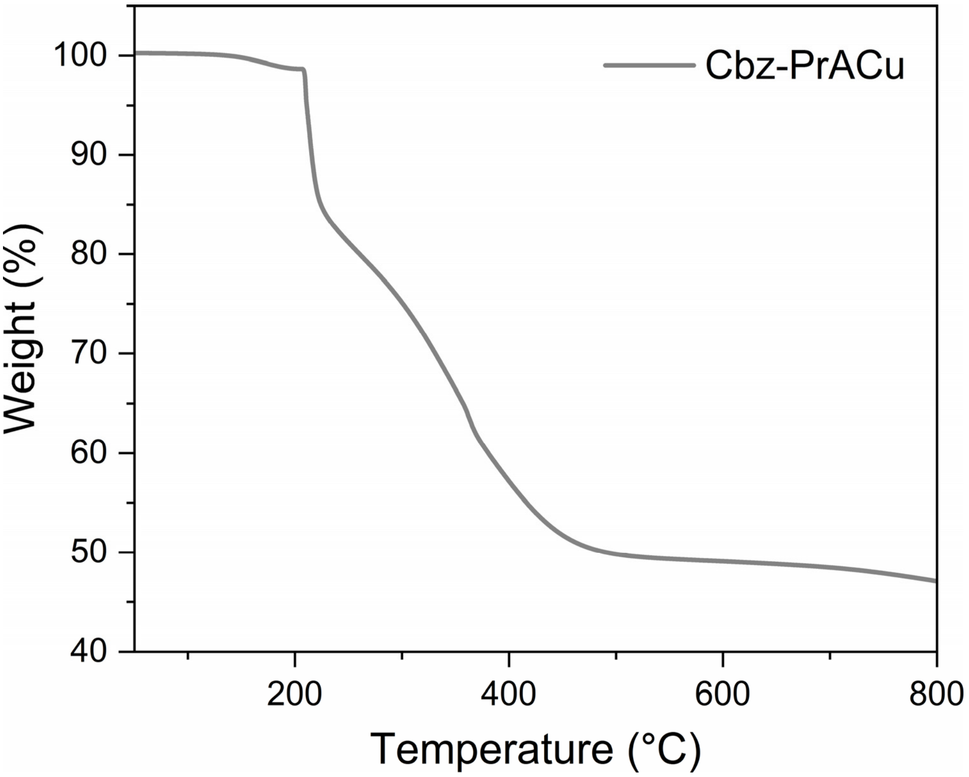

Supplementary Fig. 24 Partial density of states (PDOS) of Cu12a (a) and Cu12b (b).

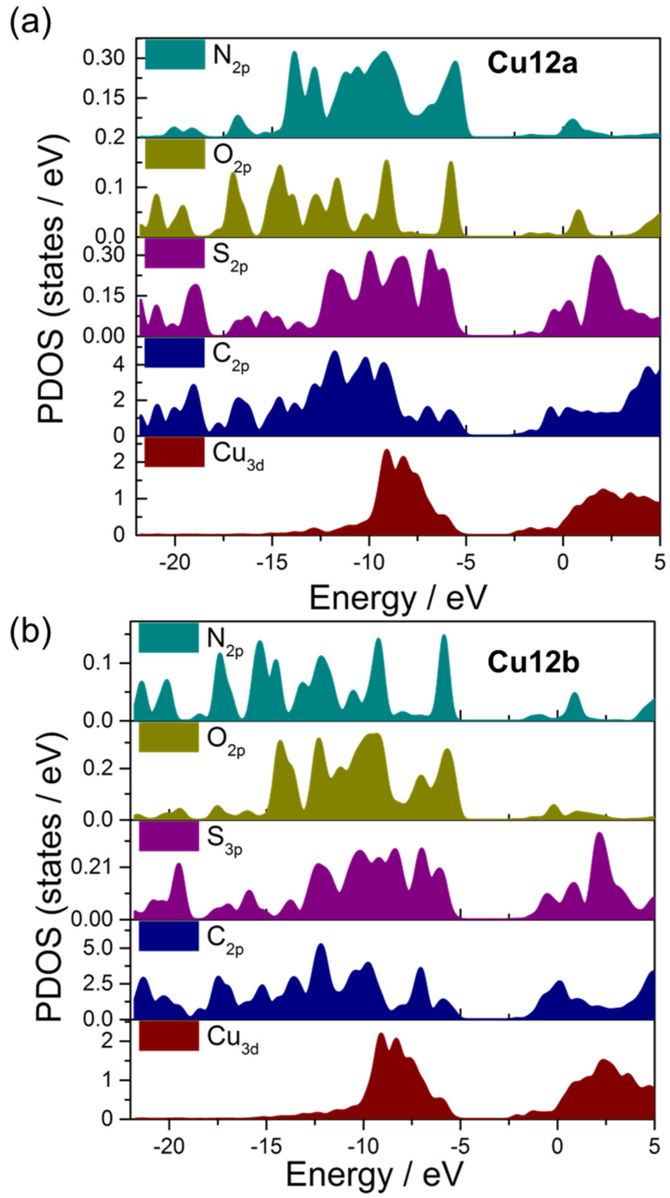

Supplementary Fig. 25 Experimental (red line) and the calculated (blue line) photon-energy plots of Cu12a (a) and Cu12b (b).

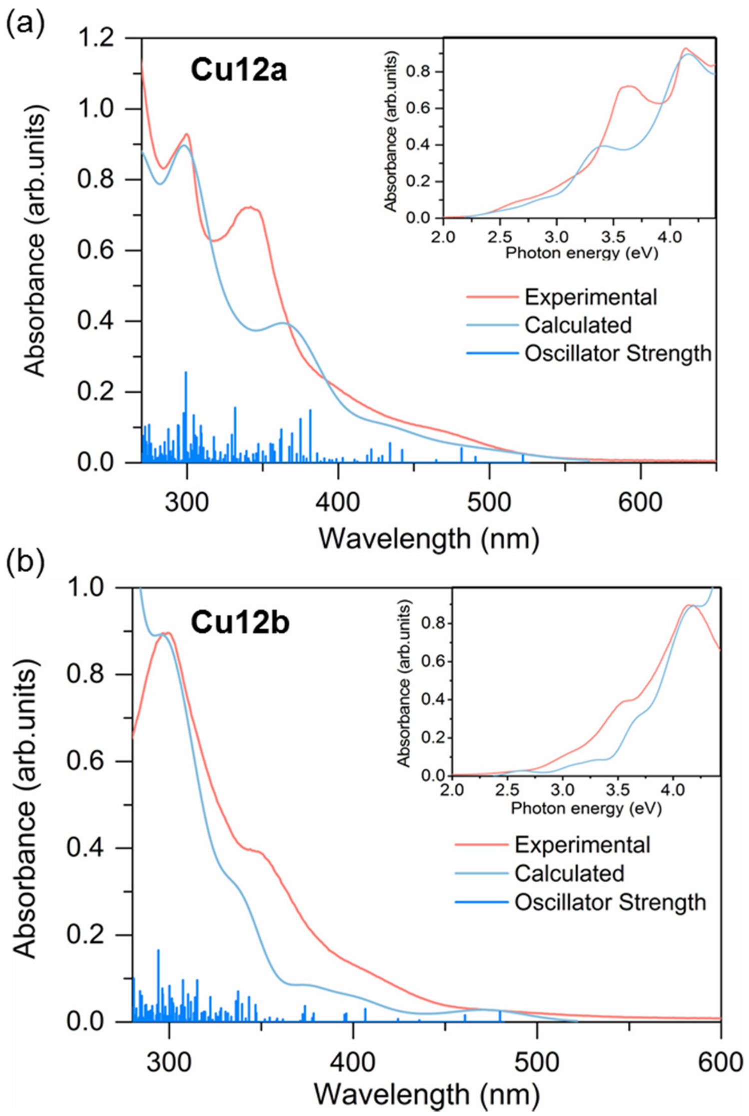

Supplementary Fig. 26 Kohn-Sham orbitals for Cu12a.

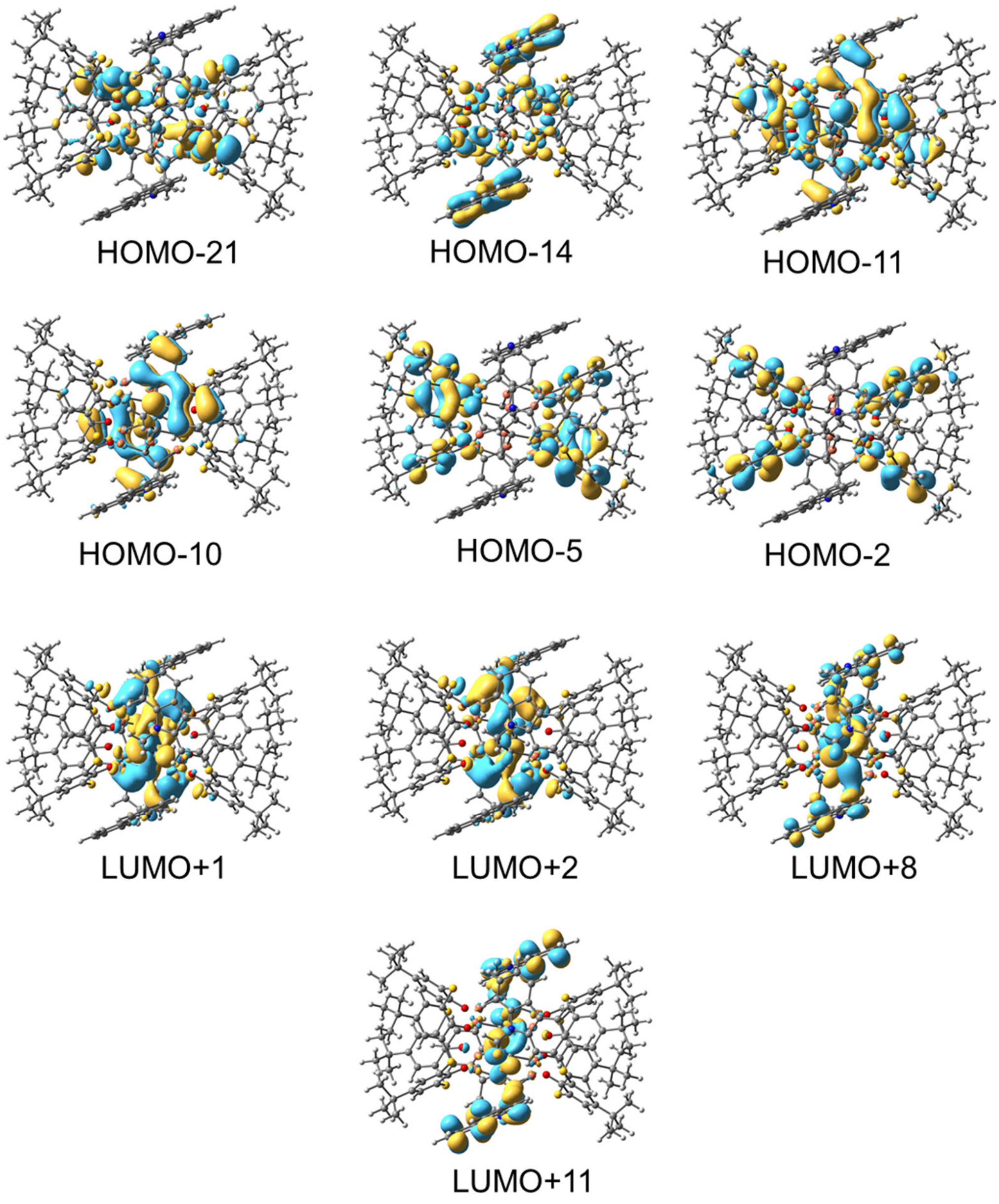

Supplementary Fig. 27 Kohn-Sham orbitals for Cu12b.

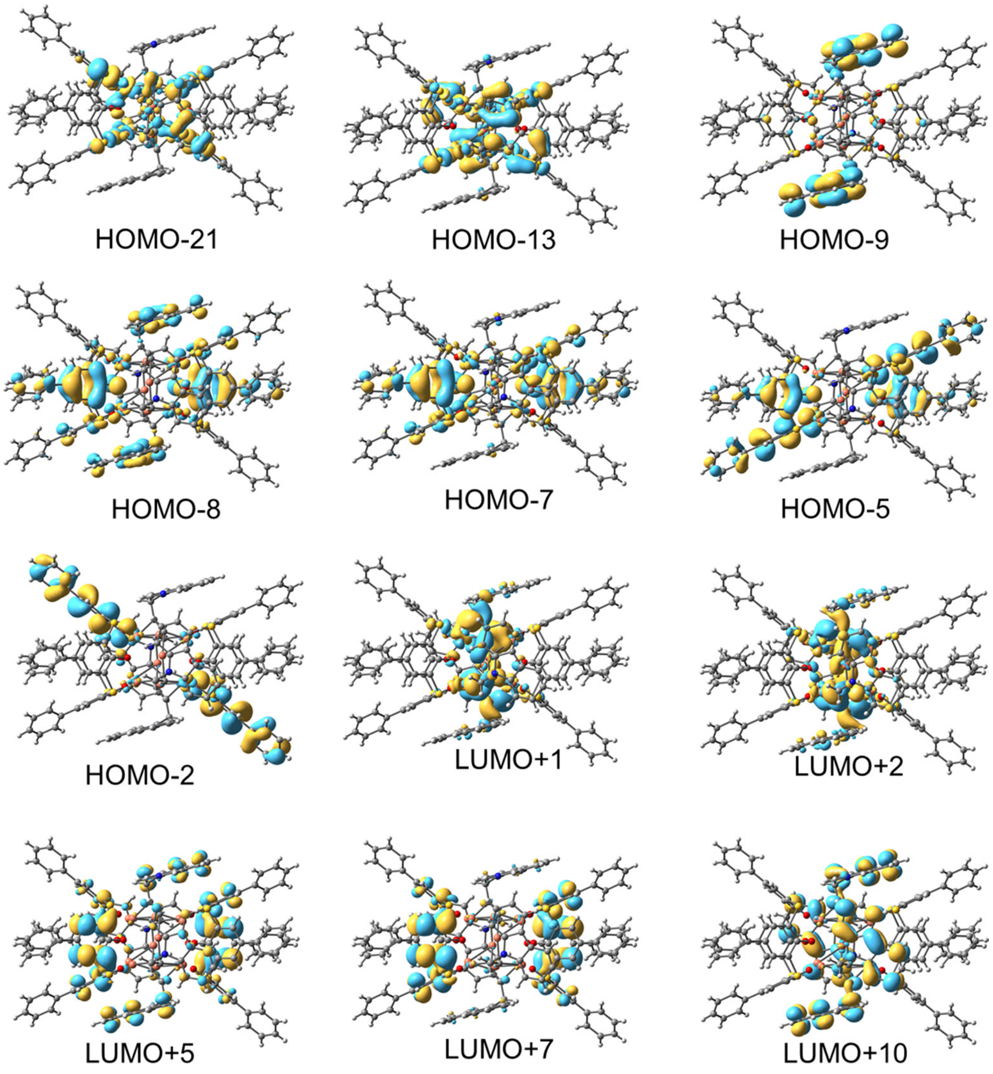

Supplementary Fig. 28 (a) Collision-induced dissociation (CID) mass spectra of Cu12a measured at the collision energy of 0–100 eV. (b) Comparison of the experimental (green line) and simulated (red line) isotopic patterns of 1f. (c) Relative intensity of the resulting 1b and 1f collected at the collision energy of 0–100 eV. (d) CID mass spectra of Cu12b measured at the collision energy of 0–100 eV. (e) Relative intensity of the resulting 2b collected at the collision energy of 0–100 eV.

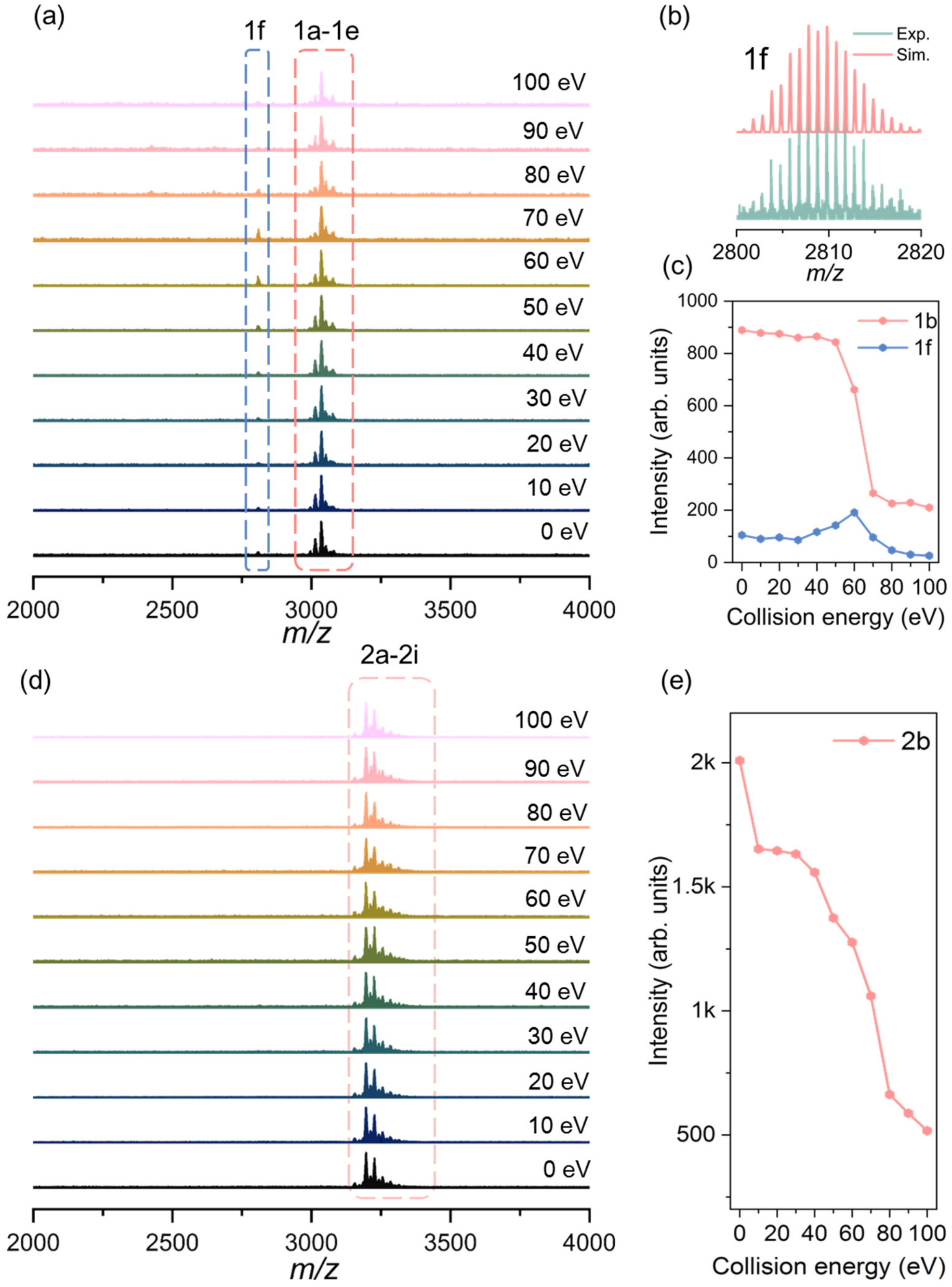

Herein, the gas-phase stabilities of two Cu_{12} clusters were studied using positive-ion mode ESI-MS with varing collision energies. For **Cu12a** (Supplementary Fig. 28a-c), the species **1a**-**1e** exhibited a consistent weakening trend as the collision energy increased from 0 to 100 eV. However, a new +1 charged species at 2807.7661 (**1f**: Cal. 2807.8198) was observed, which showed an initial increase and then a decrease in intensity. This species was identified as the fragment $[Cu_{12}(TC4A)_2(Cbz\text{-}PrA)_3]^+$, likely generated by the dissociation of one Cbz-PrA$^-$ ligand from **Cu12a**. In contrast, for **Cu12b** (Supplementary Fig. 28d-e), increasing the collision energy from 0 to 100 eV did not result in any fragmentation of the pristine cluster. This indicates that the **Cu12b** remains stable under various collision energies in the gas phase. In brief, such a CID result demonstrates the superior gas-phase stability of **Cu12b** compared to **Cu12a**.

Supplementary Fig. 29 Comparison of the experimental (green line) and simulated (red line) isotopic patterns of 3a and 2j.

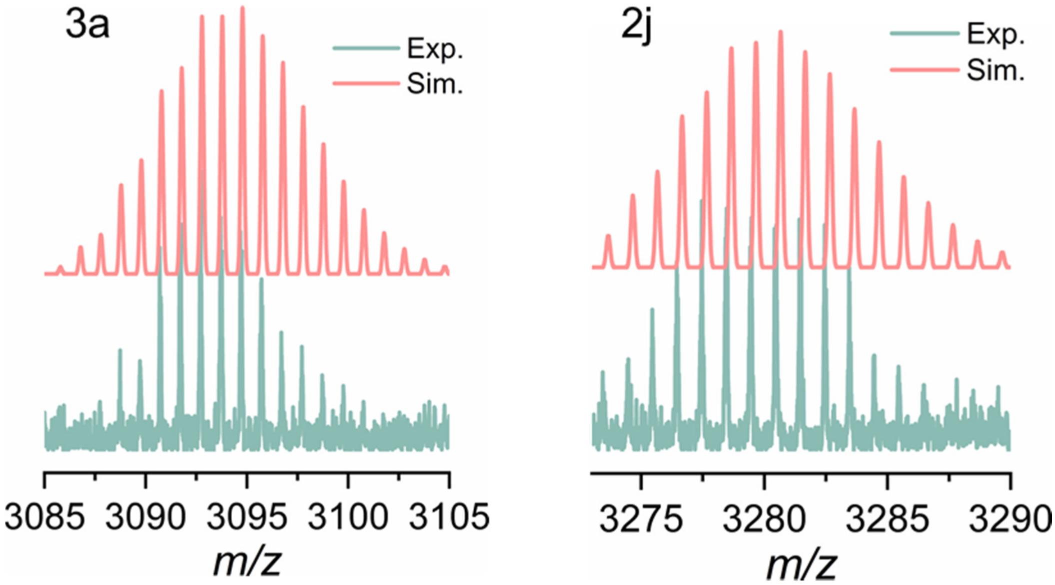

Supplementary Table 1 Crystal Data Collection and Structure Refinement.

Identification code	**Cu12a**	**Cu12a-π**	**Cu12a re-adsorption**
Empirical formula	$C_{148}H_{144}Cu_{12}N_4O_{12}S_8$	$C_{140}H_{128}Cu_{12}N_4O_8S_8$	$C_{148}H_{144}Cu_{12}N_4O_{12}S_8$
Formula weight	3189.62	3013.42	3189.76
Temperature/K	100	100	100
Crystal system	monoclinic	monoclinic	monoclinic
Space group	$P2_1/c$	$C2/c$	$P2_1/c$
a/Å	22.3420(4)	43.629(4)	22.317(2)
b/Å	13.4399(2)	13.2209(12)	13.4313(12)
c/Å	23.8733(4)	24.0350(19)	23.844(2)
α/°	90	90	90
β/°	107.6846(18)	110.114(4)	107.692(4)
γ/°	90	90	90
Volume/Å^3	6829.8(2)	13018(2)	6809.1(10)
Z	2	4	2
ρ_{calc}g/cm^3	1.551	1.538	1.556
μ/mm^{-1}	3.606	3.724	3.617
F(000)	3264.0	6144.0	3264.0
Radiation	Cu Kα (λ = 1.54184 Å)	CuKα (λ = 1.54184 Å)	Cu Kα (λ = 1.54184 Å)
2Θ range for data collection/°	7.62 to 152.726	4.314 to 130.344	7.646 to 133.94
Reflections collected	44151	61354	27143
Independent reflections	13529 [R_{int}= 0.0518, R_{sigma} = 0.0484]	11048 [R_{int}= 0.0916, R_{sigma}= 0.0659]	11834 [R_{int}= 0.0703, R_{sigma}= 0.0913]
Data/restraints/parameters	13529/159/903	11048/67/818	11834/26/874
Goodness-of-fit on F^2	1.031	1.039	1.001
Final R indexes [I>=2σ (I)]	R_1= 0.0395, wR_2= 0.0978	R_1= 0.0872, wR_2= 0.2332	R_1= 0.0681, wR_2= 0.1743
Final R indexes [all data]	R_1= 0.0475, wR_2= 0.1024	R_1= 0.1145, wR_2= 0.2568	R_1= 0.1053, wR_2= 0.2002
Largest diff. peak/hole / e Å^{-3}	1.57/-0.71	3.34/-1.05	1.05/-0.83

Identification code	**Cu12b**	**Cu12b-NACs**
Empirical formula	$C_{172}H_{128}Cu_{12}N_4O_{12}S_8$	$C_{156}H_{96}Cu_{12}N_4O_8S_8$
Formula weight	3461.74	3173.32
Temperature/K	173	100
Crystal system	triclinic	triclinic
Space group	*P*-1	*P*-1
a/Å	13.6082(4)	16.187(5)
b/Å	13.7233(3)	18.184(5)
c/Å	22.9246(6)	36.128(7)
α/°	90.480(2)	92.267(5)
β/°	100.186(2)	98.554(4)
γ/°	119.670(3)	112.286(3)
Volume/$Å^3$	3638.12(19)	9677(4)
Z	1	3
ρ_{calc}g/cm^3	1.580	1.634
μ/mm^{-1}	3.444	2.127
F(000)	1760.0	4800.0
Radiation	CuKα (λ = 1.54184 Å)	MoKα (λ = 0.71073 Å)
2Θ range for data collection/°	7.46 to 134.146	2.432 to 50
Reflections collected	36890	120673
Independent reflections	12771 [R_{int}= 0.0464, R_{sigma}= 0.0476]	34117 [R_{int}= 0.1272, R_{sigma} = 0.1251]
Data/restraints/parameters	12771/354/865	34117/702/2579
Goodness-of-fit on F^2	1.044	0.975
Final R indexes [I>=2σ (I)]	R_1= 0.0641, wR_2= 0.1946	R_1= 0.0916, wR_2= 0.2517
Final R indexes [all data]	R_1= 0.0799, wR_2= 0.2094	R_1= 0.1778, wR_2= 0.3249
Largest diff. peak/hole / e $Å^{-3}$	1.32/-0.65	1.00/-0.67

Supplementary Table 2 Selected bond distances (Å) and angles (°) for Cu12a and Cu12b.

**Cu12a**			
Cu1-Cu2^{i}	2.6379(5)	C10-C48	1.413(4)
Cu1-Cu5	2.8949(5)	C11-C13	1.527(4)
Cu1-S1	2.3699(7)	C11-C33	1.533(4)
Cu1-O1	1.9470(19)	C11-C61	1.527(5)
Cu1-O4	2.1918(18)	C11-C63	1.531(5)
Cu1-C9	2.221(3)	C12-C15	1.417(4)
Cu1-C14	1.963(3)	C12-C72	1.381(4)
Cu2-Cu3^{i}	2.4512(5)	C15-C22	1.421(4)
Cu2-Cu4	2.9365(5)	C16-C19	1.392(4)
Cu2-Cu5	2.8828(6)	C16-C24	1.391(4)
Cu2-Cu6	2.9459(5)	C17-C27	1.268(4)
Cu2-C9^{i}	1.927(3)	C17-C38	1.498(3)
Cu2-C17	2.241(3)	C18-C26	1.393(6)
Cu2-C27	2.030(3)	C18-C59	1.379(6)
Cu3-Cu5	3.0391(5)	C19-C50	1.417(4)
Cu3-S2^{i}	2.4291(7)	C20-C30	1.380(6)
Cu3-O3^{i}	2.0147(19)	C20-C32	1.400(7)
Cu3-O6^{i}	2.0326(19)	C21-C23	1.395(4)
Cu3-C9	1.941(3)	C21-C26	1.386(5)
Cu4-Cu5	2.4233(5)	C22-C39	1.387(4)
Cu4-S4^{i}	2.3675(7)	C23-C52	1.409(4)
Cu4-O1^{i}	2.1005(19)	C24-C53	1.539(4)
Cu4-O6^{i}	2.0050(19)	C24-C70	1.399(4)
Cu4-C27	1.938(3)	C25-C58	1.388(4)
Cu5-Cu6	2.5986(5)	C25-C66	1.412(4)
Cu5-C9	2.062(3)	C29-C73	1.394(4)
Cu5-C14	2.158(3)	C30-C74	1.405(5)
Cu5-C27	1.947(3)	C31-C47	1.386(5)
Cu6-S3	2.3900(7)	C31-C74	1.405(5)
Cu6-O3	2.1226(19)	C32-C47	1.395(6)
Cu6-O4	1.9460(19)	C33-C41	1.396(4)
Cu6-C17	1.968(3)	C33-C56	1.394(4)
Cu6-C27	2.258(3)	C35-C62	1.413(4)
S1-C12	1.788(3)	C35-C64	1.391(4)
S1-C37	1.792(3)	C37-C56	1.389(4)

S2-C42	1.781(3)	C37-C71	1.414(4)
S3-C19	1.791(3)	C40-C41	1.390(4)
S3-C40	1.789(3)	C40-C71	1.411(4)
S4-C22	1.791(3)	C42-C50	1.423(4)
S4-C25	1.792(3)	C42-C70	1.389(4)
O1-C15	1.316(3)	C43-C60	1.532(4)
O3-C50	1.304(3)	C44-C46	1.394(4)
O4-C71	1.322(3)	C44-C69	1.400(4)
O6-C66	1.304(3)	C45-C51	1.526(4)
N1-C7	1.447(3)	C45-C65	1.535(5)
N1-C10	1.397(4)	C45-C67	1.521(5)
N1-C35	1.391(4)	C45-C69	1.529(4)
N2-C23	1.390(4)	C46-C66	1.420(4)
N2-C31	1.396(4)	C48-C54	1.398(4)
N2-C38	1.451(4)	C48-C62	1.448(4)
C1-C53	1.547(5)	C49-C55	1.376(6)
C1A-C53	1.556(9)	C49-C62	1.401(5)
C2-C53	1.570(5)	C52-C59	1.395(5)
C2A-C53	1.499(9)	C52-C74	1.444(5)
$Cu2^{i}$-Cu1-Cu5	68.205(14)	C14-C9-Cu1	61.43(15)
S1-Cu1-$Cu2^{i}$	148.95(2)	C14-C9-$Cu2^{i}$	140.0(2)
S1- Cu1-Cu5	140.45(2)	C14-C9-Cu3	141.3(2)
O1- Cu1-$Cu2^{i}$	77.91(5)	C14-C9-Cu5	76.47(16)
O1- Cu1- Cu5	122.90(6)	N1-C10-C48	109.0(3)
O1-Cu1-S1	88.42(6)	C29-C10-N1	128.4(3)
O1-Cu1-O4	106.02(8)	C29-C10-C48	122.4(3)
O1-Cu1-C9	123.64(9)	C13-C11-C33	111.1(2)
O1-Cu1-C14	158.52(10)	C13-C11-C63	108.3(3)
O4-Cu1-$Cu2^{i}$	127.79(5)	C61-C11-C13	107.5(3)
O4-Cu1-Cu5	66.81(5)	C61-C11-C33	111.9(3)
O4-Cu1-S1	82.64(5)	C61-C11-C63	109.6(3)
O4-Cu1-C9	109.28(8)	C63-C11-C33	108.3(2)
C9-Cu1-$Cu2^{i}$	45.73(7)	C15-C12-S1	119.0(2)
C9-Cu1-Cu5	45.20(7)	C72-C12-S1	119.3(2)
C9-Cu1-S1	138.00(7)	C72-C12-C15	121.8(2)
C14-Cu1-$Cu2^{i}$	80.67(8)	Cu1-C14-Cu5	89.13(10)
C14-Cu1-Cu5	48.18(7)	C7-C14-Cu1	123.71(19)
C14-Cu1-S1	109.50(8)	C7-C14-Cu5	116.46(18)
C14-Cu1-O4	88.46(9)	C9-C14-Cu1	83.63(18)

C14-Cu1-C9	34.94(10)	C9-C14-Cu5	68.30(16)
Cu1^{i}-Cu2-Cu4	80.008(15)	C9-C14-C7	151.1(3)
Cu1^{i}-Cu2-Cu5	111.082(17)	O1-C15-C12	122.6(2)
Cu1^{i}-Cu2-Cu6	164.01(2)	O1-C15-C22	121.6(2)
Cu3^{i}-Cu2-Cu1^{i}	106.552(18)	C12-C15-C22	115.8(2)
Cu3^{i}-Cu2-Cu4	162.40(2)	C24-C16-C19	122.1(3)
Cu3^{i}-Cu2-Cu5	113.726(19)	Cu6-C17-Cu2	88.60(10)
Cu3^{i}-Cu2-Cu6	82.301(16)	C27-C17-Cu2	63.82(16)
Cu4-Cu2-Cu6	87.862(15)	C27-C17-Cu6	85.61(18)
Cu5-Cu2-Cu4	49.207(12)	C27-C17-C38	148.6(3)
C9^{i}-Cu2-Cu1^{i}	55.64(8)	C38-C17-Cu6	124.83(19)
C9^{i}-Cu2-Cu3^{i}	50.92(8)	C59-C18-C26	120.5(3)
C9^{i}-Cu2-Cu4	133.18(8)	C16-C19-S3	120.0(2)
C9^{i}-Cu2-Cu5	130.44(8)	C16-C19-C50	121.7(3)
C9^{i}-Cu2-Cu6	131.79(8)	C50-C19-S3	118.3(2)
C9^{i}-Cu2-C17	153.69(10)	C30-C20-C32	121.1(4)
C9^{i}-Cu2-C27	171.93(11)	C26-C21-C23	116.8(3)
C17-Cu2-Cu1^{i}	139.67(7)	C15-C22-S4	120.1(2)
C17-Cu2-Cu3^{i}	109.22(7)	C39-C22-S4	118.7(2)
C17-Cu2-Cu4	71.46(7)	C39-C22-C15	121.2(2)
C17-Cu2-Cu5	70.22(7)	N2-C23-C21	128.7(3)
C17-Cu2-Cu6	41.89(6)	N2-C23-C52	109.2(3)
C27-Cu2-Cu1^{i}	120.44(8)	C21-C23-C52	122.1(3)
C27-Cu2-Cu31	132.15(8)	C16-C24-C53	123.4(3)
C27-Cu2-Cu4	41.06(7)	C16-C24-C70	116.7(3)
C27-Cu2-Cu5	42.42(8)	C70-C24-C53	119.9(3)
C27-Cu2-Cu6	49.92(7)	C58-C25-S4	119.8(2)
C27-Cu2-C17	34.09(10)	C58-C25-C66	121.7(2)
Cu2^{i}-Cu3-Cu5	68.022(15)	C66-C25-S4	118.4(2)
S2^{i}-Cu3-Cu2i	147.56(3)	C21-C26-C18	122.1(3)
S2^{i}-Cu3-Cu5	139.98(2)	Cu2-C27-Cu6	86.61(10)
O3^{i}-Cu3-Cu2^{i}	83.31(6)	Cu4-C27-Cu2	95.44(11)
O3^{i}-Cu3-Cu5	128.33(5)	Cu4-C27-Cu5	77.19(10)
O3^{i}-Cu3-S2^{i}	84.75(5)	Cu4-C27-Cu6	153.11(14)
O3^{i}-Cu3-O6^{i}	105.83(8)	Cu5-C27-Cu2	92.87(11)
O6^{i}-Cu3-Cu2^{i}	127.84(5)	Cu5-C27-Cu6	75.93(9)
O6^{i}-Cu3-Cu5	66.64(5)	C17-C27-Cu2	82.09(17)
O6^{i}-Cu3-S2^{i}	84.46(5)	C17-C27-Cu4	146.5(2)
C9-Cu3-Cu2^{i}	50.42(8)	C17-C27-Cu5	136.1(2)

C9-Cu3-Cu5	42.12(8)	C17-C27-Cu6	60.33(15)
C9-Cu3-S2[i]	132.52(8)	C10-C29-C73	117.7(3)
Symmetry code: (i)1-X,1-Y,1-Z			
**Cu12b**			
Cu1-Cu2	2.9709(9)	C10-C39	1.489(7)
Cu1-Cu3	2.5997(8)	C12-C17	1.337(11)
Cu1-S4	2.3523(11)	C12-C34	1.376(11)
Cu1-O1	2.214(3)	C14-C35	1.422(6)
Cu1-O2	1.947(3)	C14-C40	1.411(7)
Cu1-C7	1.975(4)	C15-C30	1.349(11)
Cu1-C11	2.213(5)	C15-C62	1.418(10)
Cu2-Cu3	3.0446(9)	C16-C25	1.417(7)
Cu2-Cu3[i]	2.9448(9)	C16-C33	1.422(6)
Cu2-Cu4	2.5664(10)	C17-C43	1.373(10)
Cu2-Cu5	3.0252(10)	C18-C20	1.405(6)
Cu2-Cu6	2.4354(9)	C18-C27	1.415(6)
Cu2-C7	2.232(4)	C19-C24	1.407(10)
Cu2-C11	2.047(5)	C19-C59	1.378(12)
Cu2-C13	1.964(5)	C20-C52	1.389(7)
Cu3-Cu4[i]	2.9091(8)	C22-C29	1.421(7)
Cu3-Cu5	2.4397(9)	C22-C31	1.404(8)
Cu3-Cu6[i]	2.8848(10)	C24-C75	1.400(9)
Cu3-C10[i]	2.205(4)	C24-C78	1.420(10)
Cu3-C11	1.926(4)	C25-C48	1.400(7)
Cu3-C13[i]	2.024(4)	C26-C28	1.366(11)
Cu4-S1	2.3787(13)	C26-C32	1.382(11)
Cu4-O1	1.951(3)	C27-C54	1.378(7)
Cu4-O4	2.099(3)	C28-C78	1.404(9)
Cu4-C10	1.984(4)	C29-C72	1.371(7)
Cu4-C13	2.214(4)	C30-C53	1.345(11)
Cu5-S3[i]	2.4481(15)	C31-C68	1.395(7)
Cu5-O3[i]	2.019(3)	C32-C76	1.374(9)
Cu5-O4[i]	2.008(3)	C33-C41	1.387(7)
Cu5-C11	1.947(5)	C35-C56	1.387(7)
Cu6-S2[i]	2.3695(13)	C36-C65	1.315(13)
Cu6-O2[i]	2.090(3)	C36-C67	1.316(13)
Cu6-O3[i]	2.003(4)	C37-C76	1.389(9)
Cu6-C13	1.942(5)	C37-C78	1.410(9)
S1-C25	1.787(5)	C38-C8	1.3900

S1-C35	1.788(5)	C38-C69	1.3900
S2-C27	1.798(5)	C8-C45	1.3900
S2-C29	1.790(6)	C45-C21	1.3900
S3-C31	1.800(5)	C45-C70	1.488(7)
S3-C40	1.791(5)	C21-C71	1.3900
S4-C20	1.786(5)	C71-C69	1.3900
S4-C33	1.783(5)	C40-C58	1.386(7)
O1-C16	1.308(5)	C41-C60	1.386(8)
O2-C18	1.319(5)	C42-C66	1.410(8)
O3-C22	1.292(6)	C42-C77	1.393(9)
O4-C14	1.300(6)	C43-C64	1.398(9)
Cu3-Cu1-Cu2	65.87(2)	C75-N2-C39	124.7(5)
S4-Cu1-Cu2	138.67(4)	C2A-C1A-C6A	120.0
S4-Cu1-Cu3	154.73(4)	C2A-C1A-C60	120.4(9)
O1-Cu1-Cu2	64.43(8)	C6A-C1A-C60	119.6(10)
O1-Cu1-S4	82.97(8)	C4A-C3A-C2A	120.0
O2-Cu1-Cu2	122.65(9)	C3A-C4A-C5A	120.0
O2-Cu1-Cu3	77.41(9)	C6A-C5A-C4A	120.0
O2-Cu1-S4	88.30(9)	C5A-C6A-C1A	120.0
O2-Cu1-O1	104.26(14)	C1-C6-C5	120.0
O2-Cu1-C7	157.86(16)	C6-C1-C2	120.0
O2-Cu1-C11	123.67(14)	C6-C1-C60	119.0(7)
C7-Cu1-Cu2	48.70(12)	C2-C1-C60	121.0(7)
C7-Cu1-Cu3	80.74(12)	C1-C2-C3	120.0
C7-Cu1-S4	110.62(12)	C4-C3-C2	120.0
C7-Cu1-O1	89.86(15)	C5-C4-C3	120.0
C7-Cu1-C11	34.44(16)	C4-C5-C6	120.0
C11-Cu1-Cu2	43.53(12)	Cu1-C7-Cu2	89.64(16)
C11-Cu1-Cu3	46.31(11)	C11-C7-Cu1	83.2(3)
C11-Cu1-S4	141.25(12)	C11-C7-Cu2	65.0(3)
C11-Cu1-O1	106.26(14)	C11-C7-C23	150.6(5)
Cu1-Cu2-Cu3	51.19(2)	C23-C7-Cu1	124.9(3)
Cu1-Cu2-Cu5	85.36(2)	C23-C7-Cu2	117.8(3)
$Cu3^{i}$-Cu2-Cu1	106.20(3)	C64-C9-C34	120.5(7)
$Cu3^{i}$-Cu2-Cu3	83.41(3)	Cu4-C10-$Cu3^{i}$	87.81(16)
$Cu3^{i}$-Cu2-Cu5	104.23(3)	C13-C10-$Cu3^{i}$	65.0(2)
Cu4-Cu2-Cu1	83.17(3)	C13-C10-Cu4	82.8(3)
Cu4-Cu2-Cu3	112.90(3)	C13-C10-C39	151.9(4)
Cu4-Cu2-$Cu3^{i}$	63.28(2)	C39-C10-$Cu3^{i}$	116.7(3)

Cu4-Cu2-Cu5	159.58(3)	C39-C10-Cu4	124.6(3)
Cu5-Cu2-Cu3	47.40(2)	Cu2-C11-Cu1	88.35(18)
Cu6-Cu2-Cu1	157.37(3)	Cu3-C11-Cu1	77.48(16)
Cu6-Cu2-Cu3[i]	64.03(2)	Cu3-C11-Cu2	100.0(2)
Cu6-Cu2-Cu3	106.29(3)	Cu3-C11-Cu5	78.09(16)
Cu6-Cu2-Cu4	107.63(3)	Cu5-C11-Cu1	155.4(2)
Cu6-Cu2-Cu5	78.04(3)	Cu5-C11-Cu2	98.45(18)
C7-Cu2-Cu1	41.66(11)	C7-C11-Cu1	62.4(3)
C7-Cu2-Cu3	67.30(11)	C7-C11-Cu2	81.1(3)
C7-Cu2-Cu3[i]	145.97(11)	C7-C11-Cu3	139.8(4)
C7-Cu2-Cu4	111.72(12)	C7-C11-Cu5	141.9(4)
C7-Cu2-Cu5	68.75(12)	C17-C12-C34	120.8(7)
C7-Cu2-Cu6	139.32(12)	Cu2-C13-Cu3[i]	95.17(17)
C11-Cu2-Cu1	48.12(13)	Cu2-C13-Cu4	75.54(14)
C11-Cu2-Cu3[i]	121.74(12)	Cu3[i]-C13-Cu4	86.57(17)
Symmetry code: (i) 1/2-X,1-Y,1-Z			

Supplementary Table 3. Assignment of the labeled species of Cu12a and Cu12b.

Species	Charge state	Formula	Exp. (*m/z*)	Sim. (*m/z*)
**1a**	+1	$Cu_{12}(TC4A)_2(Cbz\text{-}PrA)_4H$	3014.9203	3014.9087
**1b**	+1	$Cu_{12}(TC4A)_2(Cbz\text{-}PrA)_4Na$	3036.9040	3036.8907
**1c**	+1	$Cu_{12}(TC4A)_2(Cbz\text{-}PrA)_4K$	3052.8748	3052.8645
**1d**	+1	$Cu_{12}(TC4A)_2(Cbz\text{-}PrA)_4(CH_3OH)Na$	3068.9031	3068.9169
**1e**	+1	$Cu_{12}(TC4A)_2(Cbz\text{-}PrA)_4(CH_3OH)_2H$	3078.8529	3078.9613
**1f**	+1	$Cu_{12}(TC4A)_2(Cbz\text{-}PrA)_3$	2807.7661	2807.8198
**2a**	+1	$Cu_{12}(PTC4A)_2(Cbz\text{-}PrA)_4H$	3174.6881	3174.6591
**2b**	+1	$Cu_{12}(PTC4A)_2(Cbz\text{-}PrA)_4Na$	3196.6478	3196.6410
**2c**	+1	$Cu_{12}(PTC4A)_2(Cbz\text{-}PrA)_4K$	3212.6287	3212.6149
**2d**	+1	$Cu_{12}(PTC4A)_2(Cbz\text{-}PrA)_4(CH_3OH)Na$	3228.6100	3228.6673
**2e**	+1	$Cu_{12}(PTC4A)_2(Cbz\text{-}PrA)_4(CH_3OH)K$	3244.5773	3244.6411
**2f**	+1	$Cu_{12}(PTC4A)_2(Cbz\text{-}PrA)_4(CH_3OH)_2(H_2O)H$	3256.5639	3256.7222
**2g**	+1	$Cu_{12}(PTC4A)_2(Cbz\text{-}PrA)_4(CH_3OH)_3H$	3270.6542	3270.7379
**2h**	+1	$Cu_{12}(PTC4A)_2(Cbz\text{-}PrA)_4(CH_3OH)_2(H_2O)(N_2)H$	3284.6501	3284.7283

**2i**	+1	$Cu_{12}(PTC4A)_2(Cbz\text{-}PrA)_4(CH_3OH)_4H$	3302.6969	3302.7642
**2j**	+1	$Cu_{12}(PTC4A)_2(Cbz\text{-}PrA)_4(CH_3OH)(H_2O)_2K$	3280.5150	3280.6623
**3a**	+1	$Cu_{12}(PTC4A)(TC4A)(Cbz\text{-}PrA)_4H$	3094.7467	3094.7839

TC4A: $C_{40}H_{44}O_4S_4$; PTC4A: $C_{48}H_{28}O_4S_4$; Cbz-PrA: $C_{15}NH_{10}$;

Supplementary Table 4 Comparison of iodine adsorption capacities in aqueous solution of various adsorbents.

**Materials**	**Iodine uptake capacity (g g^{-1})**	**Type**	**Reference**
**Cu12a-π**	2.96	Cluster-based PSF	This work
**C[4]P-BTP**	3.24	Calix[4]pyrrole	*Angew. Chem. Int. Ed.* **2022**, *61*, e202113724
**CaCOP3**	3.1	Calix[4]arenes	*Mater. Chem. Phys.,***2020**, *239*, 122328
**CalCOP1**	2.32	Polycalix[4]arenes	*J Mater Sci.*, **2020**, *55*, 1854
**HCOF-1**	2.1±0.1	HOF	*J. Am. Chem. Soc.* **2017**, *139*, 7172
**3D MOF-1**	1.1±0.05	MOF	*ACS Appl. Mater. Interfaces.*, **2020**, *12*, 46107
**TAPB-BPDA**	0.988	Nitrogen-containing COF	*React Funct Polym.*, **2021**, *159*, 104806
**THPS-C**	0.926	Triptycene	*Adv. Mater. Interfaces.*, **2019**, *6*, 1900249
**pSi-C**	0.25	Porous Si-C composite	*RSC Adv.*, **2021**, *11*, 5268
**G-QP6**	0.247	Quaterphen[n]arenes	*Angew. Chem. Int. Ed.* **2019**, *58*, 3885
**G-QP5**	0.245	Quaterphen[n]arenes	*Angew. Chem. Int. Ed.* **2019**, *58*, 3885
**G-TP5**	0.243	Terphen[n]arenes	*Angew. Chem. Int. Ed.* **2019**, *58*, 3885
**G-TP6**	0.24	Terphen[n]arenes	*Angew. Chem. Int. Ed.* **2019**, *58*, 3885

Supplementary Table 5 The excited states, energies (in eV and nm), oscillator strengths, weights, transitions with the strongest weights in Cu12a obtained by TD-DFT calculations.

Excitation transitions	Energy (eV)	nm	Oscillator strength (a.u.)	Weight	Orbitals	Most weighted transitions
55	3.4216	362.36	0.0472	0.3448	687-710	HOMO-10→LUMO+1 ($L_{(Cbz\text{-}PrA)}$MCT)
				0.3021	697-711	HOMO-11→LUMO+2 ($L_{(Cbz\text{-}PrA+TC4A)}$MCT)
				0.1734	687-710	HOMO-21→LUMO+1 ($L_{(TC4A)}$MCT)
				0.1712	694-710	HOMO-14→LUMO+1 ($L_{(Cbz\text{-}PrA)}$MCT)
177	4.1640	297.75	0.0703	0.2611	703-715	HOMO-5→LUMO+6 ($L_{(TC4A)}$MCT mixed ($L_{(TC4A)}L_{(Cbz\text{-}PrA)}$CT)
				0.2347	707-717	HOMO-1→LUMO+8 ($L_{(TC4A)}$MCT mixed ($L_{(TC4A)}L_{(Cbz\text{-}PrA)}$CT)
				0.1621	703-720	HOMO-5→LUMO+11 ($L_{(TC4A)}$MCT)
				0.1609	706-720	HOMO-2→LUMO+11 ($L_{(TC4A)}$MCT mixed ($L_{(TC4A)}L_{(Cbz\text{-}PrA)}$CT)

Supplementary Table 6 The excited states, energies (in eV and nm), oscillator strengths, weights, transitions with the strongest weights in Cu12b obtained by TD-DFT calculations.

Excitation transitions	Energy (eV)	nm	Oscillator strength (a.u.)	Weight	Orbitals	Most weighted transitions
49	3.6747	337.40	0.071	0.3102	731-742	HOMO-9→LUMO+1 ($L_{(Cbz\text{-}PrA)}$MCT)
				0.2852	732-743	HOMO-8→LUMO+2 ($L_{(TC4A+\ Cbz\text{-}PrA)}$MCT)
				0.1716	727-743	HOMO-13→LUMO+2 ($L_{(TC4A+\ Cbz\text{-}PrA)}$MCT)
				0.1645	735-743	HOMO-5→LUMO+2 ($L_{(PTC4A)}$MCT)
146	4.2149	294.16	0.165	0.1895	719-742	HOMO-21→LUMO+1 ($L_{(Cbz\text{-}PrA+PTC4A)}$MCT)
				0.1809	738-751	HOMO-2→LUMO+10 ($L_{(TC4A)}L_{(Cbz\text{-}PrA)}$MCT)
				0.1546	733-748	HOMO-7→LUMO+7 ($IL_{(Cbz\text{-}PrA+PTC4A)}$CT)
				0.1537	732-747	HOMO-8→LUMO+6 ($IL_{(Cbz\text{-}PrA+PTC4A)}$CT)

Supplementary Reference:

1. Rigaku Oxford Diffraction. CrysAlis^{Pro} Software system, version 1.171.40.25a, *Rigaku Corporation: Oxford, UK,* (2018).
2. Palatinus, L. & Chapuis, G. *SUPERFLIP* - A Computer Program for the Solution of Crystal Structures by Charge Flipping in Arbitrary Dimensions. *J. Appl. Crystallogr.* **40**, 786-790 (2007).
3. Sheldrick, G. M. Crystal Structure Refinement with SHELXL. *Acta Crystallogr. C: Struct. Chem.* **71**, 3-8 (2015).
4. Dolomanov, O. V., Bourhis, L. J., Gildea, R. J., Howard, J. A. K. & Puschmann, H. *OLEX2:* A Complete Structure Solution, Refinement and Analysis Program. *J. Appl. Crystallogr*. **42**, 339-341 (2009).
5. Spek, A. L. Structure Validation in Chemical Crystallography. *Acta. Crystallogr. Sect*. D. **65**, 148-155 (2009).
6. Frisch, M. et al. Gaussian 16 Rev. B. 01 (Gaussian, Inc., 2016).
7. Lu, T. & Chen, F. Multiwfn: A Multifunctional Wavefunction Analyzer. J. *Comput. Chem.* **33**, 580-592 (2012).
8. Zhang, C. et al. Solvent-Induced Isomeric Cu_{13} Nanoclusters: Chlorine to Copper Charge Transfer Boosting Molecular Oxygen Activation in Sulfide Selective Oxidation. *ACS Nano* **16**, 9598-9607 (2022).
9. Iki, N. et al. Synthesis of *p-tert*-Butylthiacalix[4]arene and its Inclusion Property. *Trahedron* **56**, 1437-1443 (2000).
10. Voss, N. R. & Gerstein, M. 3V: Cavity, Channel and Cleft Volume Calculator and Extractor. *Nucleic Acids Res.* **38**, W555-W562 (2010).
