# Supplementary material for: Dynamic and transformable Cu12 cluster-based C-H···π-stacked porous supramolecular frameworks
Source: Nat Commun. 2023 Oct 12;14:6413. doi: 10.1038/s41467-023-42201-w (PMC10570389; doi:10.1038/s41467-023-42201-w)
Supplement: Supplementary file 3 — Description of Additional Supplementary Files [file 41467_2023_42201_MOESM3_ESM.pdf]

Description of Additional Supplementary Files

Supplementary Data 1: Thermogravimetric analysis (TGA) and differential scanning calorimetry (DSC) curves of **Cu12a** and **Cu12a-π**.

Supplementary Data 2: N_2 adsorption and desorption isotherms of **Cu12a-π** at 77 K.

Supplementary Data 3: N_2 adsorption and desorption isotherms of **Cu12b-NACs** at 77 K.
